# Structural diversification during glucosinolate breakdown: mechanisms of thiocyanate, epithionitrile and simple nitrile formation

**DOI:** 10.1111/tpj.14327

**Published:** 2019-04-29

**Authors:** Daniela Eisenschmidt‐Bönn, Nicola Schneegans, Anita Backenköhler, Ute Wittstock, Wolfgang Brandt

**Affiliations:** ^1^ Department of Bioorganic Chemistry Leibniz Institute of Plant Biochemistry Weinberg 3 06120 Halle (Saale) Germany; ^2^ Institute of Pharmaceutical Biology Technische Universität Braunschweig Mendelssohnstr. 1 38106 Braunschweig Germany

**Keywords:** quantum mechanical calculation, thiocyanate‐forming protein, epithionitrile, nitrile, iron, loop structures

## Abstract

Secondary metabolism is characterized by an impressive structural diversity. Here, we have addressed the mechanisms underlying structural diversification upon damage‐induced activation of glucosinolates, a group of thioglucosides found in the Brassicales. The classical pathway of glucosinolate activation involves myrosinase‐catalyzed hydrolysis and rearrangement of the aglucone to an isothiocyanate. Plants of the Brassicaceae possess specifier proteins, i.e. non‐heme iron proteins that promote the formation of alternative products by interfering with this reaction through unknown mechanisms. We have used structural information available for the thiocyanate‐forming protein from *Thlaspi arvense* (TaTFP), to test the impact of loops protruding at one side of its β‐propeller structure on product formation using the allylglucosinolate aglucone as substrate. *In silico* loop structure sampling and semiempirical quantum mechanical calculations identified a 3L2 loop conformation that enabled the Fe^2+^ cofactor to interact with the double bond of the allyl side chain. Only this arrangement enabled the formation of allylthiocyanate, a specific product of TaTFP. Simulation of 3,4‐epithiobutane nitrile formation, the second known product of TaTFP, required an alternative substrate docking arrangement in which Fe^2+^ interacts with the aglucone thiolate. In agreement with these results, substitution of 3L2 amino acid residues involved in the conformational change as well as exchange of critical amino acid residues of neighboring loops affected the allylthiocyanate versus epithionitrile proportion obtained upon myrosinase‐catalyzed allylglucosinolate hydrolysis in the presence of TaTFP *in vitro*. Based on these insights, we propose that specifier proteins are catalysts that might be classified as Fe^2+^‐dependent lyases.

## Significance Statement

Structural diversification upon glucosinolate breakdown is controlled by specifier proteins, a group of non‐heme iron proteins. Our study provides mechanistic insights that identify specifier proteins as Fe^2+^‐dependent C‐S/C‐C lyases. By contrast with the role of Fe^2+^ in sulfur abstraction during simple nitrile formation, thiocyanate and epithionitrile formation depends on Fe^2+^/Fe^3+^ as a redox partner. Thiocyanate formation by *Thlaspi arvense* thiocyanate‐forming protein requires a conformational change that enables an alternative substrate docking pose.

## Introduction

**Figure 1:**
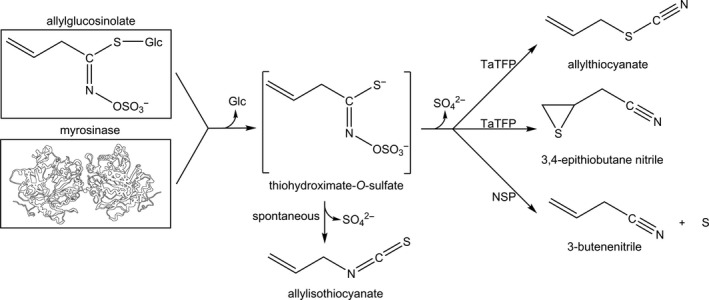
Scheme of allylglucosinolate (2‐propenylglucosinolate) activation. After plant tissue damage, myrosinase cleaves the thioglucosidic bond and an unstable thiohydroximate‐*O*‐sulfate is formed. In the presence of TFP from *T. arvense* (TaTFP) this aglucone rearranges to allylthiocyanate and epithionitrile (3,4‐epithiobutanenitrile), while a simple nitrile (3‐butenenitrile) is formed when an NSP is present. An ESP would promote only epithionitrile formation (not shown). Without specifier proteins, the aglucone undergoes spontaneous Lossen rearrangement yielding allylisothiocyanate. When TaTFP (or an ESP) is incubated with other (non‐alkenyl‐)glucosinolates and myrosinase, the simple nitrile is formed (e.g. phenylacetonitrile from benzylglucosinolate) (not shown).

Structural diversity is one of the most striking characteristics of specialized (or secondary) metabolism (Hartmann, 2007; Hamberger and Bak, 2013; Leong and Last, 2017; Owen *et al*., 2017; Pichersky and Raguso, 2018). The glucosinolate−myrosinase system of the Brassicales represents one well studied example for which the impact of natural selection on the evolution of structural diversity has been demonstrated (Benderoth *et al*., 2006; Prasad *et al*., 2012; Züst *et al*., 2012). The core of this system is formed by a group of stable and nonreactive storage molecules, the glucosinolates, and co‐occurring myrosinases (β‐thioglucoside glucohydrolase; EC 3.2.1.147) (Halkier and Gershenzon, 2006; Agerbirk and Olsen, 2012) (Figure 1). Glucosinolate activation occurs upon wounding, (e.g. due to chewing) that brings glucosinolates in contact with the myrosinases. This initiates glucosinolate hydrolysis to an unstable aglucone, which rearranges spontaneously to the corresponding isothiocyanate (Figure 1; reviewed in [Wittstock *et al*., 2016]). By contrast with glucosinolates, isothiocyanates are very reactive and toxic for microbes, nematodes, fungi, and insects (Walker *et al*., 1937; Shofran *et al*., 1998; Agrawal and Kurashige, 2003; Aissani *et al*., 2013; Jeschke *et al*., 2016). Despite the defensive potential of isothiocyanates, many species of the Brassicaceae are able to promote alternative activation pathways by the action of specifier proteins, a group of kelch domain‐containing proteins that affect the structural outcome of myrosinase‐catalyzed glucosinolate hydrolysis (Tookey, 1973; Lambrix *et al*., 2001; Wittstock and Burow, 2007; Kuchernig *et al*., 2012). In the presence of these proteins, formation of isothiocyanates is reduced in favor of alternative breakdown products, namely simple nitriles, epithionitriles, and organic thiocyanates (Figure 1). This introduces another level of structural diversification besides biosynthesis and may provide an additional layer of defense via direct or indirect effects on plant enemies (Jander *et al*., 2001; Mumm *et al*., 2008; de Vos *et al*., 2008).

Specifier proteins act specifically with respect to glucosinolate side chain and product, but do not possess hydrolytic activity on glucosinolates (reviewed in Wittstock *et al*., 2016). They are classified based on their product profile (Figure 1). Nitrile‐specifier proteins (NSPs) are the evolutionary oldest representatives, and a monophyletic origin of epithiospecifier proteins (ESPs) from NSPs has been demonstrated (Kuchernig *et al*., 2012). Phylogenetic analyses further suggest that a third group of specifier proteins, the thiocyanate‐forming proteins (TFPs), has evolved from ESPs at least twice independently (Kuchernig *et al*., 2012). A better understanding of the evolution of glucosinolate activation pathways would greatly benefit from insights into the reaction mechanisms of simple nitrile, epithionitrile, and organic thiocyanate formation and a possible catalytic role of specifier proteins. It is presently assumed that specifier proteins interact with myrosinase to capture the glucosinolate aglucone before its spontaneous conversion to an isothiocyanate. A role of iron in product formation has long been proposed based on enhanced non‐isothiocyanate product formation upon iron supplementation (Tookey, 1973; Foo *et al*., 2000; de Torres Zabala *et al*., 2005; Matusheski *et al*., 2006; Burow *et al*., 2007). The aglucone is a short‐lived intermediate that cannot be isolated for use in enzyme assays. Therefore, it has not been possible yet to provide convincing experimental evidence for an enzymatic role of specifier proteins.

One glucosinolate can give rise to up to four different breakdown products, depending on its side chain structure and the presence and type of specifier proteins. NSPs promote simple nitrile formation irrespective of the glucosinolate side chain (Burow *et al*., 2009; Kissen and Bones, 2009). ESPs have acquired the ability to produce epithionitriles upon hydrolysis of glucosinolates with a terminally unsaturated side chain, but also possess NSP activity on glucosinolates without a terminal double bond (Tookey, 1973; Lambrix *et al*., 2001; Matusheski *et al*., 2006). They share these properties with TFPs. The unique property of TFPs is their ability to also promote organic thiocyanate formation upon hydrolysis of certain glucosinolates (Gmelin and Virtanen, 1959; Burow *et al*., 2007; Kuchernig *et al*., 2011). Only three glucosinolates, namely allyl‐, benzyl‐, and 4‐methylthiobutylglucosinolate have been described to give rise to thiocyanate formation (Virtanen, 1965; Gil and MacLeod, 1980; Fenwick *et al*., 1983). TFP from *Lepidium sativum* (garden cress; LsTFP) produces thiocyanate only upon benzylglucosinolate hydrolysis while a homolog from *Thlaspi arvense* (field penny cress; TaTFP) promotes thiocyanate formation only upon allylglucosinolate hydrolysis (Burow *et al*., 2007; Kuchernig *et al*., 2011). An interesting feature of TaTFP is that it generates both allylthiocyanate and the corresponding epithionitrile, but no simple nitrile, upon allylglucosinolate hydrolysis (Kuchernig *et al*., 2011). A previous isotope labeling study demonstrated that the thiirane sulfur of epithionitriles originates from the aglucone thiolate group indicating that the thiolate sulfur is abstracted and added to the double bond during epithionitrile formation (Brocker and Benn, 1983). By contrast, experiments with isotopically labeled allylglucosinolate, TFP‐containing plant extracts and myrosinase showed that the terminal carbon atom of the aglucone side chain is linked with the thiolate sulfur during allylthiocyanate formation leading to a side chain reversion, but leaving the [S−C−N]‐moiety uncleaved (Lüthy and Benn, 1977; Rossiter *et al*., 2007). Mechanisms for these interesting chemical reactions have been discussed, but have remained unresolved (Foo *et al*., 2000; Burow *et al*., 2006; Wittstock and Burow, 2010; Brandt *et al*., 2014).

**Figure 2:**
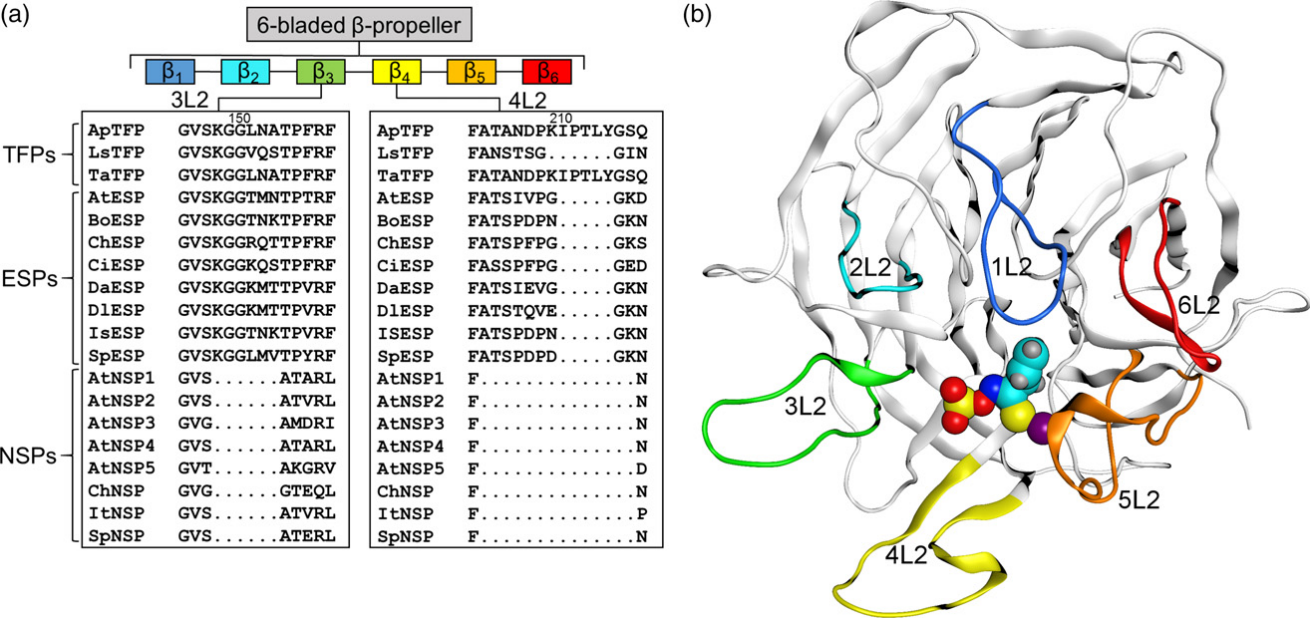
Specifier protein structure. (a) Schematic representation of the specifier protein domains with a multiple amino acid sequence alignment of 3L2 and 4L2 loops of characterized specifier proteins (for accession numbers see Table S1). (b) Side view of one TaTFP monomer with docked allylglucosinolate aglucone (spherical model, cyan C‐skeleton) and Fe^2+^ cofactor (purple sphere). Propeller blade labeling and color code in (a) and (b) are consistent with (Gumz *et al*., 2015).

Elucidation of crystal structures of TaTFP and ESP from *Arabidopsis thaliana* (AtESP) (which are homodimers), and NSP1 from *A. thaliana* (AtNSP1) (which is a monomer) and identification of an active site with a quadratic‐bipyramidally co‐ordinated iron cofactor has paved the way for detailed studies on the molecular mechanisms of product formation and the biochemical roles of specifier proteins (Brandt *et al*., 2014; Gumz *et al*., 2015; Zhang *et al*., 2016, 2017). Specifier proteins adopt a six‐bladed β‐propeller fold (Figure 2a, b). Long, blade‐connecting L4 and strand‐connecting L2 loops protrude beyond the lower propeller surface while short, strand‐connecting L1 and L3 loops are located on its upper side (Gumz *et al*., 2015). The active site is located in the central pore at the lower side of the structure and is formed largely by the L2 and L4 loops. NSPs, ESPs, and TFPs differ in the length and amino acid sequence of L2 loops of the third and fourth β‐propeller blade (3L2, 4L2; Figure 2a). This affects size and shape of their active sites (Brandt *et al*., 2014; Zhang *et al*., 2017).

Based on molecular modeling and substrate docking studies using TaTFP and allylglucosinolate aglucone as well as Ferene S‐ and Inductively Coupled Plasma Mass Spectrometry‐based iron quantification, the iron cofactor is co‐ordinated by a conserved iron‐binding triad (EXXXDXXXH; E266, D270 and H274 in TaTFP), two water molecules, and the aglucone (Brandt *et al*., 2014; Gumz *et al*., 2015; Backenköhler *et al*., 2018). Docking analysis identified an aglucone arrangement characterized by a stable interaction between the thiolate group and Fe^2+^. Conserved Arg residues (TaTFP: R94, R157) are responsible for sulfate group stabilization and nonpolar, aromatic side chains (e.g. F130 in TaTFP) are important for aglucone side chain recognition (Gumz *et al*., 2015). Despite this knowledge about geometry, Fe^2+^ dependency, and substrate binding, the reactions observed in the presence of TaTFP (or any other specifier protein) cannot be explained at the molecular level. In the present study, we address the long‐standing question on how specifier proteins amplify structural diversity during glucosinolate activation. Specifically, we used TaTFP to test what influence do flexible loop structures have on substrate binding and conversion. This turned out to be key for understanding the mechanisms of thiocyanate, epithionitrile, and simple nitrile formation and for identifying specifier proteins as catalysts.

## Results

### Changed conformation of loop 3L2 affects aglucone binding

A multiple sequence alignment of all known specifier proteins revealed that ESPs and TFPs generally possess larger 3L2 and 4L2 loops than NSPs (Figures 2 and S1) while loops 1L2, 2L2, and 6L2 are identical in length among these proteins. Moreover, the 1L2, 2L2, and 6L2 loops of TaTFP, AtESP, and AtNSP1 have similar conformations in the available 3D‐structures while loops 3L2 and 4L2 show the highest flexibility based on the B‐factors of TaTFP and AtESP (Figure S2). To study the impact of loop length and composition on TaTFP activity, we first generated, by heterologous expression of deletion constructs, mutant TaTFP proteins in which the 4L2 loop was reduced in length to that of ESPs or NSPs (TaTFP Δ210–214, TaTFP Δ204–217) or replaced by the corresponding loop amino acids of AtESP (TaTFP 205–217 × AtESP 205–212). When incubated with allylglucosinolate and myrosinase, there was no detectable specifier protein activity, likely to be due to impairment of the overall structure, and we could not draw any conclusions (Figure S3).

**Figure 3:**
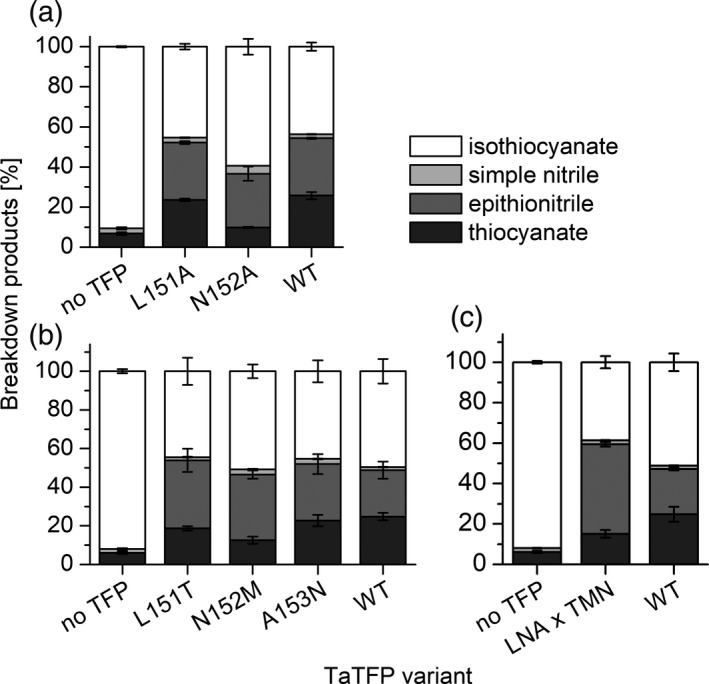
Variation of 3L2 loop residues is associated with TaTFP product profiles. (a) Replacement of L151 or N152 by Ala, (b) Replacement of L151, N152, or A153 by the corresponding residues of AtESP, (c) Replacement of the triplet L151, N152, A153 by the corresponding triplet of AtESP (LNA × TMN, triple mutant). Mutant proteins and corresponding controls (buffer only (no TFP), wild‐type [WT]) were incubated with allylglucosinolate and myrosinase in 50 mm MES buffer, pH 6.0, supplemented with 0.01 mm Fe^2+^ for 40 min. Breakdown products were quantified by GC‐FID after dichloromethane extraction. Activity is expressed as the percentage of each product relative to the total amount (nmol) of detected breakdown products. Shown are means ± SD of *N* = 3–4 independent expression experiments.

Next, we analyzed amino acid sequence variation in loop 3L2 among all known ESPs and TFPs (Figure 2a). This identified the triplet L151 N152 A153 of the 3L2 loop to be specific for TFPs with thiocyanate‐forming activity upon allylglucosinolate hydrolysis (TaTFP, ApTFP; Figure 2a). Interestingly, these amino acid residues are oriented to the protein surface at the entrance of the binding site, but they point away from the active site (Figure S4). To test their impact on product formation, we generated proteins with changed amino acid sequences by site‐directed mutagenesis of the corresponding coding sequence and heterologous expression. When we replaced L151 or N152 of the LNA triplet of TaTFP by Ala and subjected the purified mutant proteins to enzyme assays with allylglucosinolate and myrosinase, we obtained largely unchanged product proportions for TaTFP L151A when compared with wild‐type TaTFP (Figure 3a). For TaTFP N152A, activity was slightly reduced compared with wild‐type TaTFP due to a lower proportion of thiocyanate formation. To test, if TaTFP activity can be modified to resemble ESP activity through replacement of the triplet by the corresponding triplet of AtESP (T151 M152 N153), we generated the single mutants and the corresponding triple mutant (TaTFP L151T N152M A153N) and analyzed the product profiles of the purified recombinant proteins. All mutants were active, and the proportion of total non‐isothiocyanate products formed upon incubation with allylglucosinolate and myrosinase was similar to that of the wild‐type TaTFP. However, each of the three single mutants and the triple mutant produced, on average, a lower proportion of thiocyanate than wild‐type TaTFP in favor of epithionitrile formation (Figure 3b,c). The triple mutant was most strongly affected. The triplets LNA (TaTFP) and TMN (AtESP) clearly differed in their physico‐chemical properties. As the triplet pointed away from the active site and TaTFP L151A resulted in similar product proportions as wild‐type TaTFP, we hypothesized that this triplet was not directly involved in aglucone binding, but might be responsible for specific 3L2 loop conformations. These loop conformations could enable certain interactions with neighboring loops or with amino acid residues of myrosinase. The effect of the mutations on product proportions indicated that this may possibly also affect active site and/or allylglucosinolate aglucone conformation.

To investigate the impact of different 3L2 and 4L2 conformations in TaTFP (3L2: aa 144–160; 4L2: aa 204–218 [Gumz *et al*., 2015]) on allylglucosinolate aglucone binding, we applied a loop modeling approach. We performed a *de novo* loop conformation sampling using the previously described TaTFP model with docked allylglucosinolate aglucone and Fe^2+^ (PDB 5A10, [Gumz *et al*., 2015]). Additionally, we accomplished a knowledge‐based structure generation, using X‐ray structures from the deposited protein database in MOE 2016.08. Anchor triplets were defined in the flanking β‐strands of TaTFP (3L2: Y141 V142 F143/I161 E162 A163; 4L2: V198 F199 Y200/R223 V224 H225). For loop 4L2, anchor triplets were located further down‐ or upstream as the previously defined 4L2 loop region extended during the modeling procedure. For loop 3L2, 288 loop models (268 *de novo*, 20 knowledge‐based) with sufficient differences (based on a Root Mean Square Deviation [RMSD] value of >0.5 Å) were generated. A manual screening revealed that all *de novo* assembled 3L2 models were insufficient for further analysis as they exhibited large distances to the proposed TaTFP active site and the docked aglucone or intruded into the neighboring β_3_‐propeller blade. Knowledge‐based generated 3L2 loop structures were coarsely scored regarding their geometric qualities, hydrogen bonds, and van der Waals interactions and subsequently filtered with respect to their distance to the active site and interaction with the aglucone. Contrary to all other loop models, the four most promising 3L2 conformations showed C_α, L151_ to S_aglucone sulfate_ distances similar to or smaller than those determined with the previously known TaTFP model (18.26 Å, Table 1). The best evaluated 3L2 model was based on the template structure 5AXG:119–141 (Suzuki *et al*., 2015) and had a C_α, L151_ to S_aglucone sulfate_ distance of 13.04 Å.

**Table 1:** Key figures of the four most promising 3L2 models ordered according to their coarse score. The models were generated by a knowledge‐based approach and scored according to their geometry and non‐bonded interaction energies (coarse score). The distances between the C_α_ of L151 and S of the aglucone sulfate group are listed

Number	Template	Loop region	Sequence identity to TaTFP	Coarse score	Distance c_α,l151_‐s_aglucone sulfate_
1	5AXG	119–141	13.0%	−1.96	13.04 Å
2	4FCC.J	60–82	17.4%	3.51	20.46 Å
3	3T1W.A	1514–1536	17.4%	11.23	14.14 Å
4	3KEU.B	200–222	13.0%	12.66	18.84 Å

**Figure 4:**
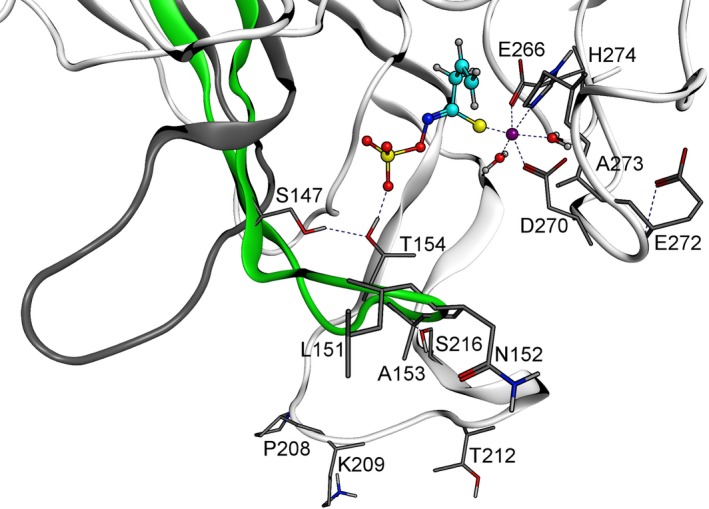
Alternative 3L2 loop conformation of TaTFP with docked allylglucosinolate aglucone and Fe^2+^. The 3L2 loop conformation identified in this study is colored in green, the previously known conformation is depicted in dark grey. Amino acids of the 3L2 (144–166), 4L2 (204–218), and 5L2 (269–283) loops (Gumz *et al*., 2015), which directly interact with Fe^2+^ or the aglucone or indirectly affect the binding site in the newly established conformation are depicted with sticks. Allylglucosinolate aglucone and Fe^2+^ are highlighted as spheres (Fe^2+^ purple, oxygen red, sulfur yellow, nitrogen blue, C‐skeleton cyan).

Finally, the geometry was optimized with Amber12:EHT in GB implicit water, and the structure was further used for a 4L2 conformation sampling. In total, 100 structurally diverse models with an RMSD value of >0.5 Å were generated (83 *de novo*, 17 knowledge‐based). However, this process did not yield an alternative 4L2 conformation to the previously described structure when the same evaluation criteria as above were applied. This result supports the validity of our conformational sampling approach, as no random assignments of new conformations were obtained. Therefore, the resulting TaTFP model possessed a previously unknown 3L2 conformation and the previously described 4L2 conformation. Loop tensions were eliminated by a short molecular dynamic simulation (NPT, 1.2 ns, 300 K) with a final energy minimization. Interestingly, a change from the previously known to the alternative 3L2 conformation upon substrate binding would act similarly to closing the lid on the active site (Figure 4).

**Figure 5:**
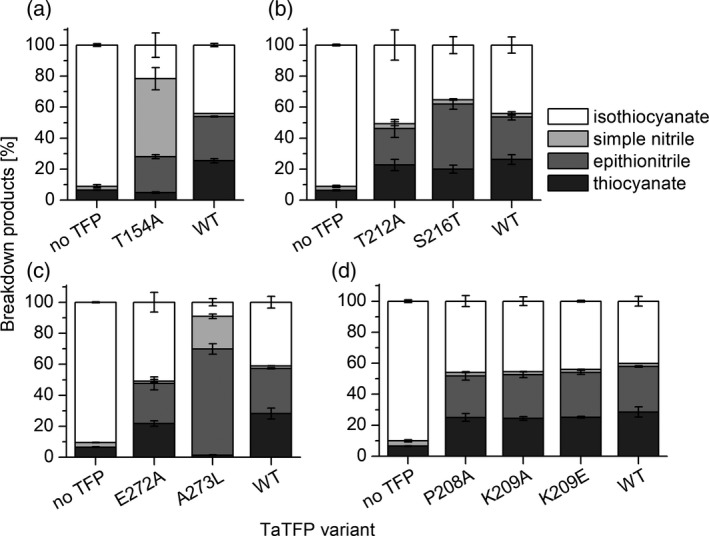
Effects of amino acid substitutions in the 3L2, 4L2, and 5L2 loops on TaTFP activity. Mutants of (a) T154, (b) T212 and S216, (c) E272 and A273, and (d) P208 and K209 and corresponding controls (buffer only (no TFP), wild‐type [WT]) were incubated with allylglucosinolate and myrosinase in 50 mm MES buffer, pH 6.0, supplemented with 0.01 mm Fe^2+^ for 40 min. Breakdown products were quantified by GC‐FID after dichloromethane extraction. Activity is expressed as the percentage of each product relative to the total amount (nmol) of detected breakdown products. Shown are means ± SD of *N* = 3 (a, d), *N* = 4 (c) or *N* = 5 (b) independent expression experiments (A273L, *N* = 3).

The alternative 3L2 conformation enabled an interaction of T154 of the 3L2 loop with one of the sulfate group oxygens of the allylglucosinolate aglucone and with S147 via hydrogen bonds. In support of a role of T154 in product formation, exchange of T154 by Ala through site‐directed mutagenesis of the corresponding coding sequence yielded an active protein with modified product profile. While wild‐type TaTFP formed simple nitrile only at background levels when incubated with allylglucosinolate and myrosinase, TaTFP T154A produced more than 50% simple nitrile (relative to total product amount) at the expense of thiocyanate and epithionitrile (Figure 5a). Thiocyanate formation was more strongly affected than epithionitrile formation. The mutant protein was highly active as indicated by a high proportion of total non‐isothiocyanate products (i.e. reduced level of isothiocyanate released by myrosinase) in the presence of TaTFP T154A as compared with TaTFP wild‐type (Figure 5a). Therefore, T154 appears to be critical for thiocyanate and epithionitrile formation by TaTFP, which underpins our modeling results. Its replacement by Ala does not generally hinder binding of the allylglucosinolate aglucone to the active site, but the conformation required for epithionitrile and especially thiocyanate formation might be less favored.

To experimentally challenge our alternative TaTFP model, we analyzed a series of mutant proteins with amino acid substitutions in the 3L2, 4L2, and 5L2 loops. Among several amino acid exchanges introduced to the 4L2 loop of TaTFP, only S216T affected the product profile (Figure 5b,d). In the presence of TaTFP S216T, myrosinase‐catalyzed hydrolysis of allylglucosinolate yielded a higher proportion of epithionitrile at the expense of mostly isothiocyanate than in the presence of wild‐type TaTFP. This reinforced the result of our 3L2 loop conformation sampling, i.e. the alternative TaTFP model (Figure 4) in which S216 forms a hydrogen bond to the T154 backbone nitrogen. This stabilizes the ‘closed lid’ conformation, which might be required for thiocyanate formation. The slightly larger Thr residue is still able to support this conformation but generates more room in favor of the aglucone conformation that allows epithionitrile production. By contrast, substitution of A273 of loop 5L2, which lines the central pore of TaTFP at the opposite side of T154, with Leu resulted in a highly active epithionitrile‐ and simple nitrile‐producing protein that lacked the ability to form thiocyanate (Figure 5c). We assume that the room‐filling Leu residue prevents the conformational change supposedly required for thiocyanate production. It is likely to generate more space in the active site, allowing not only the epithionitrile‐forming reaction, but even simple nitrile formation to take place. An exchange of the neighboring E272, which is also located at the protein surface (loop 5L2), by Ala did not affect product formation (Figure 5c). Similarly, substitution of P208, K209, or T212 of the 4L2 loop by Ala did not result in changed specificity, despite their position at the surface around the central pore of TaTFP (Figure 5b,d). Even reversion of side chain charge at position 209 (K209E) did not affect product profile or activity (Figure 5d). These residues are probably not directly involved in substrate binding. The amino acid substitutions do not seem to hinder the assumed conformational change and/or allow for alternative binding arrangements without an impact on product formation.

**Figure 6:**
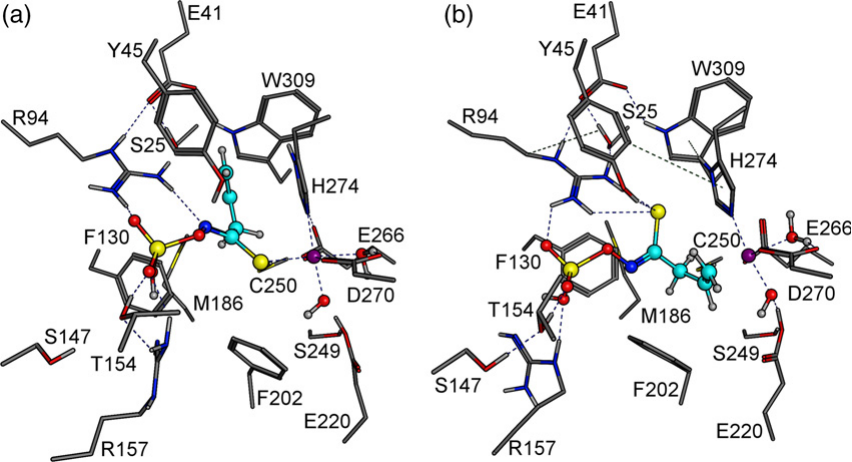
Different docking positions of allylglucosinolate aglucone into TaTFP active site. (a) Pose I, known from previous studies and characterized by thiolate‐Fe^2+^ co‐ordination. (b) Pose II with a terminal ethene group‐Fe^2+^ co‐ordination, identified after 3L2 and 4L2 conformation sampling. Fe^2+^ purple, oxygen red, sulfur yellow, nitrogen blue, aglucone C‐skeleton cyan.

Next, we were interested in the question why the proposed 3L2 loop conformational change might be a prerequisite for allylthiocyanate formation by TaTFP. As the 3L2 loop conformational change is likely to be associated with a changed set of amino acids exposed for interactions with the substrate, we repeated the GOLD docking of allylglucosinolate aglucone into the alternative TaTFP model. Fifty docking arrangements were created and evaluated with GOLD Score and calculated binding affinities. Several poses showed sufficient substrate stabilization based on the previously defined docking criteria (see above). Most of these were among the top 10 poses according to GOLD Score and calculated binding affinity. The most favored docking arrangement (pose I) is shown in Figure 6(a).

**Figure 7:**
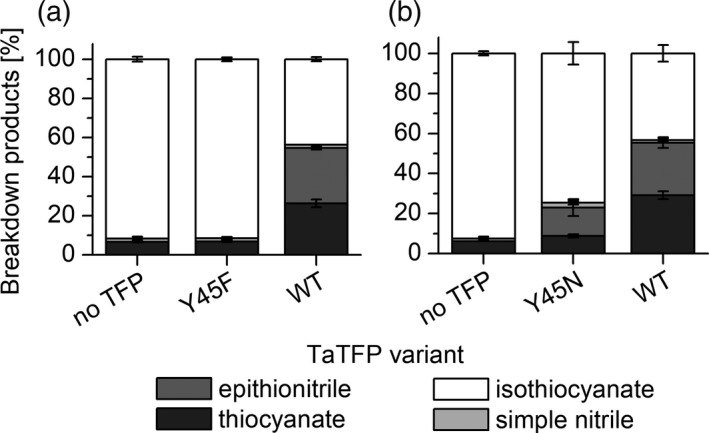
Effects of substitutions of Y45 on TaTFP activity. Y45 was replaced by (a) Phe or (b) Asn. Mutants and corresponding controls (buffer only (no TFP), wild‐type [WT]) were incubated with allylglucosinolate and myrosinase in 50 mm MES buffer, pH 6.0, supplemented with 0.01 mm Fe^2+^ for 40 min. Breakdown products were quantified by GC‐FID after dichloromethane extraction. Activity is expressed as the percentage of each product relative to the total amount (nmol) of detected breakdown products. Shown are means ± SD of *N* = 4 independent expression experiments.

Therefore, the alternative 3L2 conformation in TaTFP does not seem to prevent aglucone binding. However, we searched the full set of 50 poses for unusual, previously unknown arrangements that could possibly be associated with TaTFP product specificity. Surprisingly, we found four aglucone arrangements within the TaTFP active site in which Fe^2+^ was co‐ordinated by the terminal ethene group of the allyl side chain (instead of the aglucone thiolate group in the previously known arrangement). Only one of these poses, pose II (Figure 6b), ranked among the top 20 based on the calculated binding affinities, likely to be because of the relatively high uncertainty with which the GOLD Score fitness function describes and evaluates metal−carbon co‐ordination (Seebeck *et al*., 2008). Furthermore, the distances between involved carbon atoms and Fe^2+^ were in the range 2.5 Å to 2.8 Å, i.e. slightly larger than an ideal co‐ordination bond. To circumvent this problem, soft distance restraints between 2.0 Å and 2.4 Å with a scale factor of five were defined to tether the atoms during the subsequent geometry optimization with Amber12:EHT. Besides the Fe^2+^‐ethene group co‐ordination, the optimized protein−ligand complex exhibited a previously undiscovered thiolate group stabilization by Y45 and R94 and a strong interaction between R94 and R157 and the aglucone sulfate moiety (Figure 6b). This may explain the previous observation that TaTFP R94A, when compared with wild‐type TaTFP, produces a higher proportion of epithionitrile as well as considerable amounts of the simple nitrile (not produced by wild‐type) at the expense of the thiocyanate in enzyme assays with allylglucosinolate and myrosinase (Gumz *et al*., 2015). When we replaced Y45 by Phe, TaTFP activity was lost (Figure 7a). Although we cannot exclude effects of the mutation on the backbone structure, this result points at the importance of the hydroxyl group of Y45. Replacement with a polar residue (TaTFP Y45N) restored some of the activity, but only to a little extent (Figure 7b). Taken together, loop conformation search and molecular modeling of allylglucosinolate aglucone binding to TaTFP identified an alternative conformation of the 3L2 loop and allows unusual, previously undiscovered aglucone arrangements.

### Conformational change is required for TaTFP‐catalyzed allylthiocyanate formation

Lüthy and Benn and later on Rossiter *et al*. detected a side chain rearrangement of the allylglucosinolate aglucone during allylthiocyanate formation (Lüthy and Benn, 1977; Rossiter *et al*., 2007). To reproduce this rearrangement in semiempirical quantum mechanical calculations and to identify the role of TaTFP during the reaction, we applied a variety of semiempirical scans (one reaction co‐ordinate) and two‐dimensional grid calculations with PM7 in MOPAC 2016. To test if a certain Fe^2+^ co‐ordination or a Fe^2+^/Fe^3+^ redox reaction is essential for product formation, we used the two contrasting allylglucosinolate aglucone arrangements, pose I and pose II, obtained by the docking approach (Figure 6a,b). As the atom order of S, C, and N is preserved during thiocyanate formation, we compared the two arrangements with respect to the destabilizing effects on the C−S bond. First, the aglucone arrangement in pose II was more stable (ΔH_f_ = −5280.63 kJ mol^‐1^) than that in pose I (ΔH_f_ = −5214.10 kJ mol^‐1^). Second, hydrogen bond formation between Y45 and R94 and the thiolate group observed in pose II (Figure 6b) destabilized the C−S bond to a lesser extent than the Fe^2+^−thiolate interaction seen in pose I. The lower C−S destabilization in pose II may prevent fast bond cleavage (as it occurs during epithionitrile formation). Furthermore, the Fe^2+^ co‐ordination of the double bond resulted in a polarization of the π‐electronic system of the double bond leading to a partially positive (δ^+^) metal‐averted side and enabled a nucleophilic attack of S to C3. The charge displacement also affected neighboring carbon atoms and potentially debilitated the C−C1 bond which has to be cleaved during the rearrangement.

**Figure 8:**
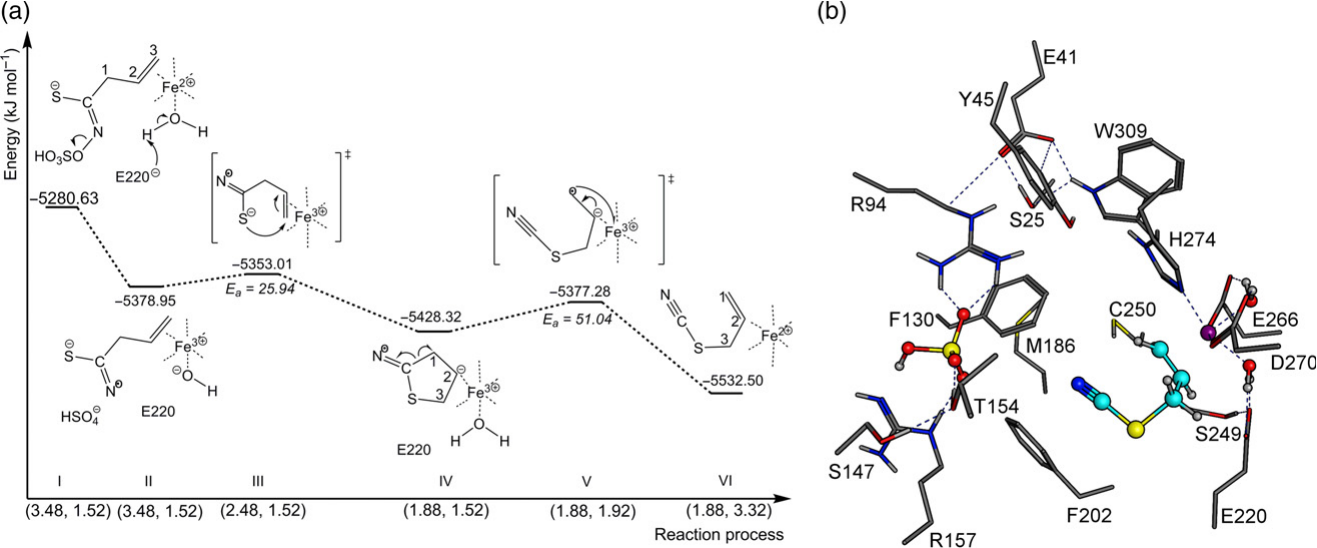
TaTFP‐catalyzed formation of allylthiocyanate. (a) Energy profile of allylthiocyanate formation. Numbers on *x*‐axis are the C3−S and C−C1 distances in Å. Each reaction step is described by corresponding heat of formations. Total reaction enthalpy is Δ_r_H_f_ = −251.87 kJ mol^−1^ (see Movie S1 for an animated presentation). (b) Optimized geometry of active site residues at the final reaction step. Fe^2+^ purple, oxygen red, sulfur yellow, nitrogen blue, aglucone C‐skeleton cyan, and amino acid C‐skeleton in grey.

To simulate the rearrangement for allylthiocyanate production in PM7 calculations starting with pose II, the elongation of the C−C1 bond and a contraction of the S−C3 distance were defined as reaction co‐ordinates (Figure 8a and Movie S1). The reaction started with spontaneous removal of the sulfate group protonated by R157 (Figure 8a I to II) with an energy release of Δ_r_H_f_ = –98.32 kJ mol^−1^. A single electron transfer to N, due to Fe^2+^/Fe^3+^ oxidation, stabilized the intermediate structure (Figure 8a II). Additionally, one of the Fe^3+^‐co‐ordinating water molecules dissociated to OH^‐^ and H^+^, and the proton was gathered by E220 (which is highly conserved among TFPs, ESPs, and NSPs; Figure S1). This step contributed to the first energy release. Convergence of the thiolate and C3 introduced a conformational change of the aglucone side chain. An activation barrier of E_a_ = 25.94 kJ mol^−1^ had to be passed to arrange S and the δ^+^ side of the double bond perpendicular to each other leading to an orbital overlap (Figure 8a III). At this transition state the S−C3 distance was 2.47 Å and the C‐C1 bond length 1.5 Å. Exceeding the transition state resulted in a changed Fe^3+^ co‐ordination. A cyclic intermediate with a formally formed C2 carbanion (Figure 8a IV) donates its free electron pair into the metal complex. Simultaneously, E220 deprotonated and restored the co‐ordinating water. The second transition state was reached at a C−C1 distance of 1.88 Å with an activation energy of E_a_ = 51.04 kJ mol^−1^. This represented the rate‐determining step of allylthiocyanate formation (Figure 8a V) and was followed by a homolytic C−C1 bond cleavage. Finally, allylthiocyanate was formed with an energy release of Δ_r_H_f_ = −155.22 kJ mol^−1^ while still bound to Fe^3+^ (Figure 8b). When allylthiocyanate dissociated from the metal complex, the donated electron pair was used for double bond formation and the single electron reduced Fe^3+^ to Fe^2+^ (Figure 8a VI).

**Figure 9:**
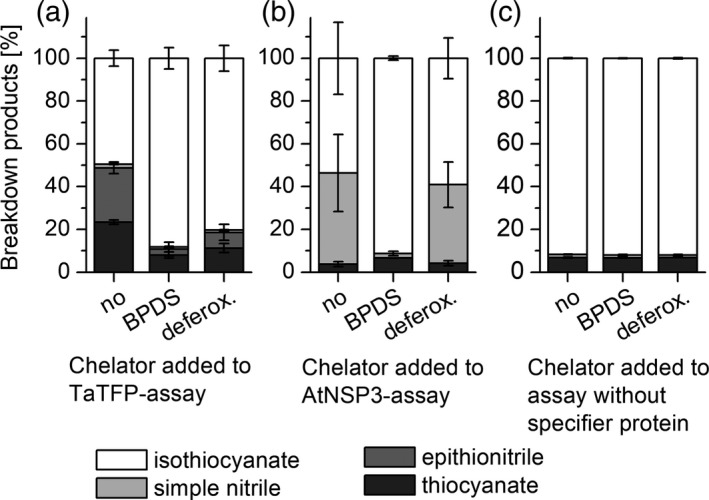
Effect of chelators on TaTFP and AtNSP3 activity. Allylglucosinolate was incubated with (a) TaTFP, (b) AtNSP3, or (c) without specifier protein in 50 mm MES buffer, pH 6.0, supplemented with 0.01 mm Fe^2+^, 5 mm BPDS, 5 mm deferoxamine or no chelator, and myrosinase for 40 min. Breakdown products were quantified by GC‐FID after dichloromethane extraction. Activity is expressed as the percentage of each product relative to the total amount (nmol) of detected breakdown products. Shown are means ± SD of *N* = 3 independent expression experiments.

Previous work showed that supplementation of TaTFP activity assays with Fe^2+^, but not Fe^3+^, led to increased product formation by TaTFP (Backenköhler *et al*., 2018). Therefore, we tested if the involvement of the redox pair Fe^2+^/Fe^3+^ predicted above can be confirmed experimentally. We compared product formation upon incubation of allylglucosinolate with TaTFP and myrosinase in the presence of bathophenanthroline disulfonate (BPDS), a chelator of Fe^2+^, or deferoxamine, a chelator of Fe^3+^ (Figure 9a). Supplementation of reactions with either of these chelators strongly reduced alternative product formation by TaTFP, i.e. thiocyanate and epithionitrile formation (Figure 9a). For thiocyanate formation, activity was reduced almost to background levels. The effect of BPDS was slightly stronger than that of deferoxamine. The results are in agreement with participation of both Fe^2+^ and Fe^3+^ in thiocyanate and epithionitrile formation.

A simulation of allylthiocyanate formation starting with pose I was unsuccessful. In most calculations with pose I, the dissociation of the thiolate group was one of the first steps. This indicates that Fe^2+^ significantly destabilized the corresponding C−S bond. Therefore, only with pose II a simulation of allylthiocyanate formation with reasonable reaction energies was possible (Movie S1). As this docking pose depends on a conformational change of the 3L2 loop upon aglucone binding, this confirms that a conformational change of the 3L2 loop is required for allylthiocyanate formation.

### TaTFP catalyzes epithionitrile formation

**Figure 10:**
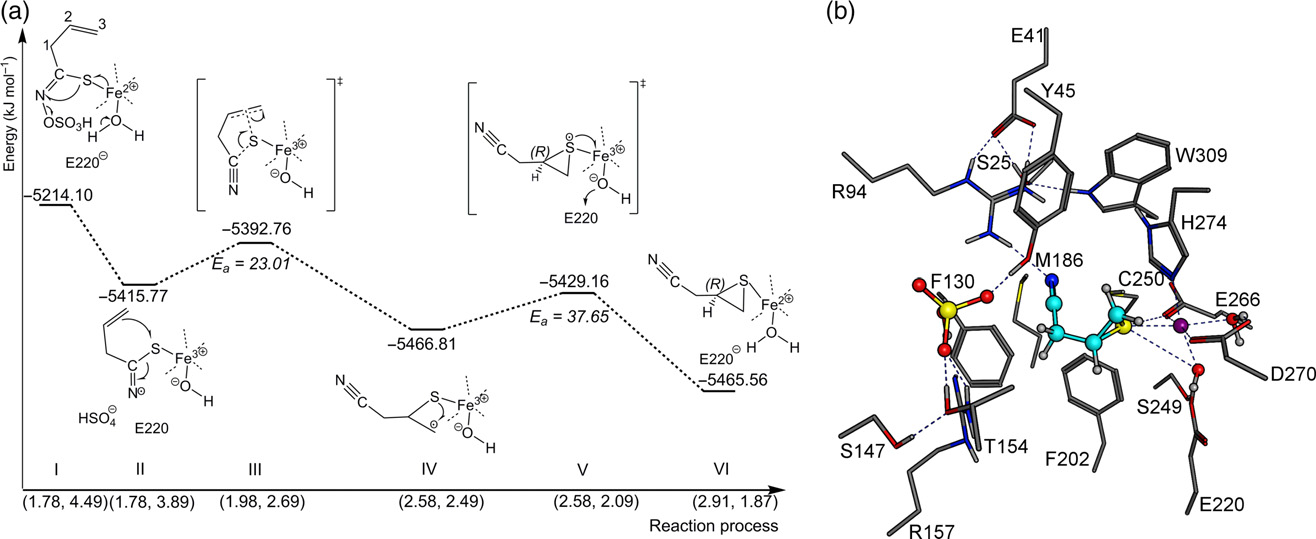
TaTFP‐catalyzed formation of *R*‐epithionitrile. (a) Energy profile of *R*‐epithionitrile formation. Numbers on *x*‐axis are the C−S and C3−S distances in Å. Each reaction step is described by corresponding heat of formations. Total reaction enthalpy is Δ_r_H_f_ = −251.46 kJ mol^−1^ (see Movie S2 for an animated presentation). (b) Optimized geometry of active site residues at the final reaction step. Fe^2+^ purple, oxygen red, sulfur yellow, nitrogen blue, aglucone C‐skeleton cyan, and amino acid C‐skeleton in grey.

Since docking pose I (Figure 6a) appeared to enable C−S bond destabilization of the allylglucosinolate aglucone (see above), a detailed investigation of TaTFP‐catalyzed epithionitrile formation was done starting with this docking pose (animated in Movie S2). A reaction between the thiolate and the terminal double bond of the allylglucosinolate aglucone was simulated with semiempirical grid calculations. Elongation of the C−S bond and distance reduction between C3 and S were defined as reaction co‐ordinates. The first steps of this reaction sequence were similar to those observed during allylthiocyanate formation. This included dissociation of the sulfate group protonated by R157 (Δ_r_H_f_ = −201.61 kJ mol^−1^ [Figure 10a I to II]), water dissociation and protonation of E220, and single electron transfer from Fe^2+^ to N to stabilize the intermediate (Figure 10a II). A rearrangement of the intermediate structure led to a conformational change of adjacent amino acid side chains which further explains the resulting energy release. The existing bilateral −I effect caused by the electron‐pulling nitrogen, and the S−Fe^3+^ co‐ordination reduced C−S bond stability. A further S−C3 distance reduction resulted in a conformational change of the allyl side chain and convergence of sulfur and terminal double bond. The first transition state (Figure 10a III) was reached with a moderate activation energy of E_a_ = 23.01 kJ mol^−1^ and described the electron distribution between adjacent atoms (C2, S, C) and homolytic opening of the C−S and the C2−C3 bonds. The small distances between S and C2 as well as S and C with 1.86 Å and 1.98 Å, respectively, promoted this rearrangement. According to localized orbital analysis (NBO), N lost its radical character and a nitrile group is formed. Next, a C2−S bond was formed (Figure 10a IV) with an energy release of Δ_r_H_f_ = −74.05 kJ mol^−1^. The convergence of the sp^3^‐hybridized C3 radical and the sulfur atom resulted in an activation barrier of E_a_ = 37.65 kJ mol^−1^ (Figure 10a V). The transition state was characterized by distances between C and S of 2.58 Å and between S and C3 of 2.09 Å, respectively. Next, the thiirane ring of *R*‐epithionitrile closed with an energy release of Δ_r_H_f_ = −36.4 kJ mol^−1^ (Figure 10a, b). The single electron located on S reduced Fe^3+^ to Fe^2+^. The deprotonation of E220 rebuilt the Fe^2+^ complex, so that TaTFP is able to bind another aglucone (Figure 10a VI). Involvement of a Fe^2+^/Fe^3+^ redox reaction in epithionitrile formation is in agreement with our experimental data that demonstrated that supplementation of TaTFP activity assays with either BPDS or deferoxamine resulted in strongly reduced epithionitrile formation by TaTFP (Figure 9a, see above).

Although one would expect stereospecificity for an enzymatic reaction, AtESP has been proposed to catalyze the formation of *R‐* and *S*‐epitionitrile during allylglucosinolate breakdown in a ratio of 1:1 based on GC‐MS using a chiral phase (Daxenbichler *et al*., 1968; Burow *et al*., 2006). When we analyzed products of allylglucosinolate breakdown by myrosinase and TaTFP, we obtained almost the same result (Figure S5).

In an attempt to explain the formation of a racemic mixture, we performed corresponding PM7 calculations based on pose I and an alternative docking pose of the aglucone (Figure S6). Details of these results and a hypothetical explanation for the formation of a racemic mixture are presented in Figure S7 and discussed in Appendix S1.

### AtNSP3‐catalyzed allylcyanide formation

**Figure 11:**
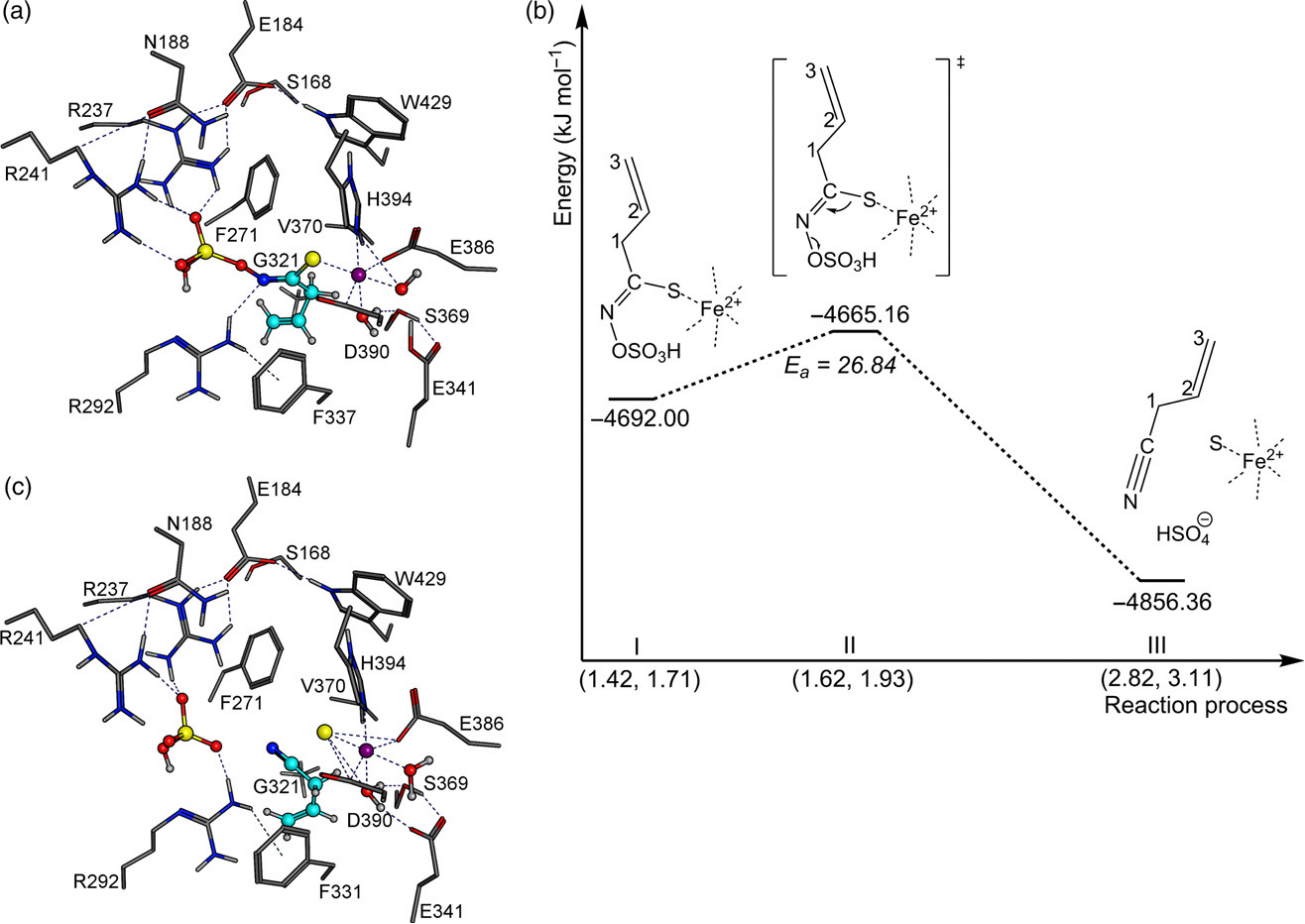
AtNSP3‐dependent allylcyanide formation. (a) Most favored allylglucosinolate aglucone orientation. (b) Energy profile of allylcyanide formation. Numbers on *x*‐axis are the N−O and C−S distances in Å. Each reaction step is described by corresponding heat of formations. Total reaction enthalpy is Δ_r_H_f_ = −164.36 kJ mol^−1^ (see Movie S3 for an animated presentation). (c) Optimized geometry of the active site at the final reaction step. Fe^2+^ purple, oxygen red, sulfur yellow, nitrogen blue, aglucone C‐skeleton cyan, and amino acid C‐skeleton in grey.

As known TFPs and ESPs do not produce simple nitriles when incubated with allylglucosinolate and myrosinase, we investigated the formation of the simple nitrile (3‐butenenitrile, allylcyanide) from allylglucosinolate aglucone using the structure of AtNSP3 with docked aglucone (Figure 11a) (Backenköhler *et al*., 2018). All known NSPs, including AtNSP3, have shorter 3L2 and 4L2 loops than TaTFP and AtESP (see above) resulting in a more open binding site and higher flexibility of the aglucone. To investigate AtNSP3‐catalyzed formation of allylcyanide on a semiempirical level, the N−O bond was elongated to simulate sulfate group dissociation. Simultaneously, the C−S bond was enlarged to reproduce a bond cleavage (Figure 11b). At an N−O bond length of 1.62 Å and a C−S bond length of 1.93 Å (Figure 11b II) the sulfate group, protonated by R292, dissociated with an activation barrier of Ea = 26.84 kJ mol^−1^. At the same time, the C−S bond was cleaved and allylcyanide was formed with an energy release of Δ_r_H_f_ = −191.20 kJ mol^−1^ (Figure 11b III, 11c). The entire reaction is displayed in Movie S3.

Potential electron transfers were investigated by localized bond orbital analysis. Contrary to TaTFP‐dependent epithionitrile and thiocyanate formation, no oxidation/reduction of the iron cofactor took place. This indicates that, for allylcyanide formation by AtNSP3, the Fe^2+^ cofactor is not involved as redox partner, but is important for intensifying the existing charge displacement within the aglucone core structure. To test this experimentally, we supplemented AtNSP3 activity assays with BPDS or deferoxamine. By contrast with the results obtained with TaTFP, product formation by AtNSP3 was affected only by BPDS and not by deferoxamine (Figure 9b). This confirms the importance of Fe^2+^ for simple nitrile formation without a role as redox partner.

## Discussion

As the active site of specifier proteins is located within the flexible loops protruding at the lower side of the β‐propeller (Gumz *et al*., 2015), loop structures are likely to impact substrate binding and conversion. The available crystal structures represent only one of the possible conformations of each loop, but B‐factors indicate high flexibility, especially of the 3L2 and 4L2 loops. Therefore, in order to understand the protein‐substrate interactions and the mechanisms of product formation by specifier proteins, a more comprehensive consideration of loop conformations is required and accurate loop structures have to be determined. This poses a challenge to *in silico* investigations, especially because there are several loops, and they may interact with each other. Based on loop conformation search and molecular dynamics simulations performed with the previously described structure of TaTFP, we identified an ‘open’ and a ‘closed’ conformation of loop 3L2. The ‘closed’ conformation enables an alternative aglucone pose at the active site. While the previously known docking pose of the allylglucosinolate aglucone in TaTFP is characterized by a thiolate‐Fe^2+^ co‐ordination, the Fe^2+^ interacts with the terminal ethene group of the allyl side chain in the alternative pose described here. Only this pose, but not the previously known one, allows the allylglucosinolate aglucone to react to allylthiocyanate in semiempirical quantum mechanical calculations (Figure 8). The alternative docking pose has never been observed in previous studies with the ‘open’ 3L2 conformation. Therefore, we proposed that a conformational change to the ‘closed’ form is required for allylthiocyanate formation by TaTFP. By contrast, epithionitrile formation by TaTFP does not seem to require this conformational change and the alternative docking pose. Starting with the previously known pose, the thiolate−Fe^2+^ co‐ordination destabilizes the C−S bond. As a consequence, the bond is cleaved and the sulfur incorporated into the terminal double bond (Figure 10). Our results suggest that the ability of TaTFP to bind the allylglucosinolate aglucone in two different ways with respect to Fe^2+^ cofactor co‐ordination is an essential precondition for its broad product profile. The inductive effect of Fe^2+^ affects the aglucone on specific sites and guides the reaction in a specific direction. The predicted energy profiles (Figures 10 and S6) demonstrate the principal ability of TaTFP to produce both enantiomers of the epithionitrile in agreement with the experimental data (Figure S5).

The *in silico* results are well supported by mutational analysis of TaTFP. Amino acid substitutions within the 3L2, 4L2, and 5L2 loops that we expected to disturb the ‘closed’ conformation (T154A, S216T, A273L; Figure 5) led to a strong to moderate reduction of the proportion of allylthiocyanate in favor of simple nitrile and/or epithionitrile formation. Substitutions of neighboring residues to T154 with the corresponding residues of AtESP also shifted product proportions in favor of the epithionitrile (L151, N152, A153; Figure 3b,c). T154 is conserved among all known TFPs and ESPs, but replaced by Ala in NSPs. Therefore, besides this residue itself, the overall loop structure and/or its interactions with surrounding loops appear to be critical for the specific product profile obtained upon glucosinolate hydrolysis. One central element of TaTFP‐catalyzed allylthiocyanate and epithionitrile formation is the oxidation of Fe^2+^ to Fe^3+^ to stabilize the intermediate structure after sulfate group dissociation. Consequently, capture of either Fe^2+^ or Fe^3+^ by BPDS and deferoxamine, respectively, strongly reduced TaTFP activity (Figure 9). This has also been reported before for epithionitrile formation by AtESP (Burow *et al*., 2006). The formation of the simple nitrile by AtNSP3 did not require Fe^2+^ oxidation in semiempirical quantum mechanical calculations (Figure 11). The dissociation of the sulfate group resulted in a spontaneous C−S bond cleavage and simple nitrile formation. In agreement with this and a previous report on simple nitrile formation by an insect NSP, which is structurally unrelated to plant specifier proteins (Burow *et al*., 2006), AtNSP3 activity assays were not affected by supplementation with deferoxamine, a chelator of Fe^3+^ (Figure 9). Therefore, our results suggest that the charge displacement caused by the thiolate‐Fe^2+^ co‐ordination in AtNSP3 is sufficient for simple nitrile formation. A directed reaction to epithionitrile or thiocyanate does not seem to be possible because of the large and open binding site of NSPs that potentially allows aglucone binding with different unspecific side chain conformations. The fate of the released sulfur is unresolved. It might be recycled in anabolic pathways of other sulfur containing metabolites (Falk *et al*., 2007) or accumulate as elemental sulfur, a known defense compound (Williams *et al*., 2002). In fact, elemental sulfur has been reported as a product of simple nitrile formation (Benn, 1977).

Future research should study in more detail, if the conformational change of the TaTFP 3L2 loop is initiated by interaction with the substrate, by an interaction with myrosinase or both. In addition, knowledge about the spin state of Fe^2+^/Fe^3+^ during the reaction would help to better understand the processes in the TaTFP active site. The conversion of other substrates, e.g. aglucones of other aliphatic or benzylic glucosinolates, by TaTFP should also be investigated. Furthermore, it has to be tested if other specifier proteins including AtESP and LsTFP function in a similar way as TaTFP and AtNSP3 and if their substrate and product specificity can be explained by the structure of their active sites.

Based on the insights into the reaction mechanisms of TaTFP and AtNSP3, we propose to ascribe a catalytic role to specifier proteins. Their function includes: (i) providing and positioning the Fe^2+^ cofactor required for the formation of thiocyanate, epithionitrile, and simple nitrile; (ii) establishing a protein environment that favors specific aglucone conformations; and (iii) protonation and deprotonation of intermediates such as protonation of the aglucone sulfate and deprotonation of one of the Fe^2+^‐co‐ordinating water molecules for thiocyanate and epithionitrile formation by TaTFP. Based on the presented reaction mechanisms, specifier proteins could be classified as metal‐dependent lyases with C−S lyase or C−S/C−C lyase activity. As the evolutionary oldest group, NSPs use the Lewis acidity of Fe^2+^ to facilitate a heterolytic bond cleavage between the central carbon and the electron‐donating sulfur. This Fe^2+^‐dependent C−S lyase activity has been retained in ESPs and TFPs, but has been extended by the ability to oxidize Fe^2+^ and to transfer its electron to the reaction intermediate. This impedes simple nitrile formation and enables a new reaction, i.e. epithionitrile formation. For TaTFP, we found that enlargement of the 3L2 loop is associated with higher flexibility and enables an alternative loop conformation that is prerequisite for an additional activity as C−C lyase resulting in thiocyanate formation. From an evolutionary point of view it is interesting that the structure of the active site has become more confined during the evolution of specifier proteins. Starting from NSPs with a large and open active site and relative low specificity, changes of the loop structures lead to more restricted aglucone conformations in ESPs and TFPs and must have provided selective advantage to the plants by allowing new products to be formed upon glucosinolate hydrolysis. Maybe simple nitrile formation acted mostly as a route of glucosinolate activation that circumvents harmful isothiocyanates to be released to plant tissue and provided sulfur for primary metabolism or as a defense while epithionitriles and thiocyanates evolved as new defense compounds. However, physiological roles of simple nitriles, epithionitriles and thiocyanates or their possible defensive functions are not well understood today and deserve further attention.

Taken together, molecular modeling, mutational analysis and semiempirical quantum mechanical calculations enabled us to propose mechanisms of simple nitrile, epithionitrile and thiocyanate formation by specifier proteins that are in agreement with a catalytic role of specifier proteins as Fe^2+^‐dependent lyases. This sheds light on the evolution of metabolic diversity in an activated plant defense system.

## Materials and methods

### Molecular modeling and loop conformation sampling

Molecular modeling was carried out using structure PDB 5A10 (TaTFP with docked allylglucosinolate aglucone and Fe^2+^; Gumz *et al*., 2015) and protein model ma‐r8uy2 (https://www.modelarchive.org/; Backenköhler *et al*., 2018; AtNSP3). Multiple sequence alignment of all 19 specifier proteins was performed with the BLOck SUbstitution Matrix (BLOSUM) 62 in MOE 2016.08 (Molecular Operating Environment 2016.08, Chemical Computing Group Inc., Montreal, QC, Canada). The penalty for gap opening was 10 and for gap extension 2. Alignments of all 3L2 and 4L2 loops were isolated manually. The knowledge‐based and *de novo* loop conformation sampling was carried with the loop modeler tool in MOE 2016.08. Only X‐ray structures with a resolution of >2.5 Å were considered during the knowledge‐based approach. Generated loop models with a C_α_‐RMSD value of <0.5 Å to each other and an energy deviation of >100 kcal mol^−1^ to the global energy minimum were discarded. Loop models were evaluated regarding their calculated coarse score, their distances to the proposed active site and their orientation to the adjacent β‐propeller blade. The geometry optimization and energy minimization of the changed TaTFP protein structure was performed with the Amber12:EHT force field (Gerber and Müller, 1995) (Case *et al*., 2012) in a GB implicit water model. For a subsequently short molecular dynamic simulation TaTFP was inserted into a spherical simulation box, filled with explicit water molecules and neutralized by NaCl counter ions. The NPT simulation was done at 300 K, 1.2 nsec (200 psec equilibration, 1000 psec production) using the Nosé−Poincaré−Andersen (NPA) equation of motion (Bond *et al*., 1999; Sturgeon and Laird, 2000).

### Protein−ligand docking

Docking of the allylglucosinolate aglucone into TaTFP and AtNSP3 was done in GOLD (Verdonk *et al*., 2003; Hartshorn *et al*., 2007) using the GOLD Score fitness functions for first evaluation. Amino acids inside a radius of 15 Å around the C_ζ_ atoms of TaTFP R94 and AtNSP3 R237 guanidine groups formed the active site. For TaTFP side chains of Y45, R94, F130, T154, R157 and S216 were flexible according the GOLD rotamer library. In AtNSP3 side chain flexibility of N188, F271, R237, R292 and H394 was considered. Fe^2+^ was described as an octahedral co‐ordinated cofactor. Water molecules involved in the binding site were always present during the docking procedure. In both cases 50 different docking positions were generated. Resulting protein−ligand complexes were energy minimized with Amber12:EHT in GB implicit water and their interaction energies regarding TaTFP and AtNSP3 were calculated. Pictures containing the entire protein structure or parts of it were created with MOE 2016.08 and further processed with GNU Image Manipulation Program (GIMP 2.8.10).

### Semiempirical calculations

During the evaluation of the completely unknown catalytic mechanisms many alternative reaction routes had to be studied. To reduce computational time we decided to apply semiemprical calculations using PM7 (Stewart, 2013) in the Molecule Orbital PACkage (MOPAC) 2016 (version 17.231 L) (Stewart, 1990, 2016) including all amino acid residues of the active site. Therefore, we accepted slight deviations, in particular of calculated transition state energies, from values that might have been obtained by more advanced methods, e.g. molecular dynamics simulations based on DFT‐QM/MM calculations, that would allow access to e.g. small entropy contributions. However, the average unsigned error in heats of formations for PM7 was given with 17.2 kJ mol^−1^ (http://openmopac.net/MOPAC2012brochure.pdf) confirming its suitability for our purpose. To study the reaction mechanisms for simple nitrile formation in AtNSP3 and epithionitrile and allylthiocyanate production in TaTFP, the proteins were reduced to their particular active sites. Therefore, 17 amino acids of TaTFP active site and 16 amino acids of AtNSP3 located in the first and second sphere around the bound aglucone were selected for calculations. To avoid unexpected side effects of the specifier protein backbone atoms only amino acid side chains were used. To keep the positions of all C_α_ atoms, such as in the entire protein structure, they were fixed during all semiempirical calculations. For optimization, the Broyden−Fletcher−Goldfarb−Shanno (BFGS) algorithm (Broyden, 1970; Fletcher, 1970; Goldfarb, 1970; Shanno, 1970) was used. To allow a potential electron transition during the reaction an open shell system was defined using UHF. For the ground state of both systems, a singlet multiplicity was assumed. Performed scan and grid calculations were performed with a step size of ± 0.2 Å. For each reaction co‐ordinate (scan)/pair of reaction co‐ordinates (grid), the final heat of formation (ΔH_f_) of the system was calculated directly resulting in an energy profile (scan) or an energy hyperplane (grid) from which the corresponding energy pathways had to be extracted. Transition state analysis was realized by fine‐mesh scans and grids with a step size of ±0.05 Å. Investigation of the electronical behavior during the reactions, including potential Fe^2+^/Fe^3+^ transition and a radical formation was carried out with Natural Bond Orbital (NBO, LOCALIZE) analysis (Von Niessen, 1972), bond order (BONDS), and partial charges (1SCF) interpretation (Armstrong *et al*., 1973; Perkins and Stewart, 1982). The SMOOTH keyword was used to circumvent calculation artefacts potentially arising through the order of changing affected co‐ordinates. Grids and scans were analyzed in MOE 2016.08 using a set of svl‐scripts implemented by Richard Bartelt. (Basic Molecular Tools, MOE GUI for MOPAC2012 scan/grid calculations, https://svl.chemcomp.com/filedetails.php?lid=1004&cid=37). All energy profiles were graphically created in ChemDraw 15.0.

### Generation of expression constructs

The expression construct described in Kuchernig *et al*. (2011) for production of recombinant TaTFP with an N‐terminal Strep‐Tag II was used to express TaTFP in *Escherichia coli* and to generate constructs for expression of mutant proteins. For AtNSP3 expression, the construct described by Backenköhler *et al*. (2018) was used. Deletions and point mutations were introduced to the *TaTFP* open reading frame by PCR on a Biometra TProfessional basic or gradient thermocycler using the primers listed in Table S2. Reactions were set up in a total volume of 25 μL *Pfu* PCR buffer and contained 10 pmol of each primer, 0.3 mm dNTPs (Thermo Scientific), 1.25 U *Pfu* DNA polymerase (Thermo Scientific) or *Pfu* Cx Hotstart DNA polymerase (Agilent), and 100 ng template. For deletion constructs, PCR fragments were generated with the following temperature program: 95°C for 3 min, 35 cycles of 95°C for 45 sec, appropriate annealing temperature (55–72°C) for 1 min, and 72°C for 1 min and a final incubation at 72°C for 10 min. PCR products were introduced into USER‐modified pET52b(+) (Novagen) (Kuchernig *et al*., 2011). For site‐directed mutagenesis, the following temperature program was used: 95°C for 3 min, followed by one cycle of 95°C for 45 sec, appropriate annealing temperature (55–72°C) for 1 min, and 72°C for 13 min, 10 cycles of this type in which the annealing temperature was reduced by 0.3°C per cycle, 13 cycles of 95°C for 45 sec, 56–72°C for 1 min, and 72°C for 13 min and a final incubation at 72°C for 10 min. This was followed by digestion with 10 U *Dpn*I (Thermo Scientific) per 19 μL PCR reaction at 37°C for 1 h. An aliquot of the reaction was used to transform *E. coli* XL1Blue MRF’ (Stratagene), plasmids were isolated and sequenced. To obtain the expression constructs, the USER cassette was excised from the plasmids by digestion with *Xba*I and *Eco*RI and transferred to *Xba*I/*Eco*RI digested USER‐modified pET52b(+) (Novagen) (Kuchernig *et al*., 2011). All expression constructs were confirmed by sequencing (Eurofins MWG operon, Ebersberg, Germany).

### Heterologous expression, purification and analysis of wild‐type and mutant TaTFP and AtNSP3

For each expression experiment, *E. coli* BL21(DE3) pLysS (Invitrogen, Germany) was freshly transformed with expression constructs for TaTFP or AtNSP3 wild‐type and mutants. Single colonies selected on Luria–Bertani (LB) medium containing 100 μg mL^−1^ ampicillin and 34 μg mL^−1^ chloramphenicol were transferred to 20 mL terrific broth (TB) containing the same antibiotics. Cultures were grown at 18°C 200 rpm for 48 h and used to initiate expression cultures. Expression cultures were grown at 18°C and 200 rpm, induced by addition of 1 mm isopropyl‐1‐thio‐β‐d‐galactopyranoside (IPTG) at an A_600_ of 0.35–0.5 and grown for another 16 h (wild‐type and mutant TaTFP) or 48 h (AtNSP3). Cells were pelleted and extracted as described by Gumz *et al*. (2015). Extracts were loaded onto Strep‐Tactin Sepharose (wild‐type and mutant TaTFP) or Strep‐Tactin XT (AtNSP3) for purification of the recombinant proteins according to the instructions by the manufacturer (IBA, Göttingen, Germany). The elution buffer was composed as follows: 2.5 mm desthiobiotin, 150 mm NaCl, 100 mm Tris‐HCl, pH 8 (for Strep‐Tactin Sepharose) or 50 mm biotin, 150 mm NaCl, 100 mm Tris−HCl (for Strep‐Tactin XT). Purity of the eluted recombinant proteins was analyzed by SDS‐PAGE. Protein concentrations were determined with the BCA Protein Assay Kit (Thermo Scientific) according to the manufacturer's instructions using bovine serum albumin (BSA) as a standard.

Enzyme assays were performed as described previously (Kuchernig *et al*., 2011). Briefly, purified recombinant protein (30 μg in 100 μl elution buffer, unless otherwise stated) or 100 μl elution buffer (negative control) was incubated with 2 mm allylglucosinolate (AppliChem), 0.01 mm (NH_4_)_2_Fe(SO_4_)_2_ and 0.005 units myrosinase (Kuchernig *et al*., 2011) purified from *Sinapis alba* seeds based on Burow *et al*. (2006) in 50 mm MES buffer, pH 6, in a total volume of 500 μL at 22°C. After 40 min, 50 μL of phenylcyanide (Sigma‐Aldrich, 100 ng μL^−1^ in MeOH) were added and breakdown products were extracted with dichloromethane and analyzed by GC using an Agilent 6890N series gas chromatograph (Agilent, Waldbronn, Germany) as described previously (Kuchernig *et al*., 2011). Quantification was based on GC‐FID peak areas, effective carbon numbers and response factors (RF) relative to the internal standard. Effective carbon numbers and RF were as follows (Gumz *et al*., 2015): allyl‐CN 4.1 (RF 1.54), allyl‐NCS 4.7 (RF 1.34), allyl‐SCN 4.1 (RF 1.54), 2,3‐epithiopropyl‐CN (3,4‐epithiobutanenitrile) 3.3 (RF 1.91). Based on the amount in nmol, the relative amounts were calculated as a percentage of the total amount (nmol) of detected glucosinolate breakdown products. Proteins represented in the same graph were analyzed in parallel. The effects of BPDS (Sigma) and deferoxamine (Sigma) were tested at 5 mm in reaction mixtures composed as described above.

## Supporting information


**Figure S1.** Multiple sequence alignment of TaTFP, AtESP, and AtNSP3.

**Figure S2.** B‐factors of TaTFP and AtESP X‐ray structures.

**Figure S3.** Effects of 4L2 loop deletions on TaTFP activity.

**Figure S4.** 3L2 conformation of TaTFP X‐ray structure (PDB 5A10).

**Figure S5.** *R*/*S*‐3,4‐epithiobutane nitrile formation in TaTFP‐containing reaction mixtures.

**Figure S6.** TaTFP‐catalyzed formation of *S*‐epithionitrile.

**Figure S7.** Energy cycles for the formation of *R*‐ and *S*‐epithionitrile.

**Table S1.** Known specifier proteins.

**Table S2.** Primers used for mutagenesis.

**Appendix S1.** Hypothetical explanation for the formation of a racemic mixture of *R*/*S*‐3,4‐epithiobutane nitrile by TaTFP

**Movie S1.** TaTFP‐catalyzed thiocyanate formation.

**Movie S2.** TaTFP‐catalyzed epithionitrile formation.

**Movie S3.** AtNSP3‐catalyzed allylcyanide formation.
