# Supplementary figures and images for: Structural diversification during glucosinolate breakdown: mechanisms of thiocyanate, epithionitrile and simple nitrile formation
Source: Plant J. 2019 Apr 29;99(2):329–43. doi: 10.1111/tpj.14327 (PMC6850609; doi:10.1111/tpj.14327)

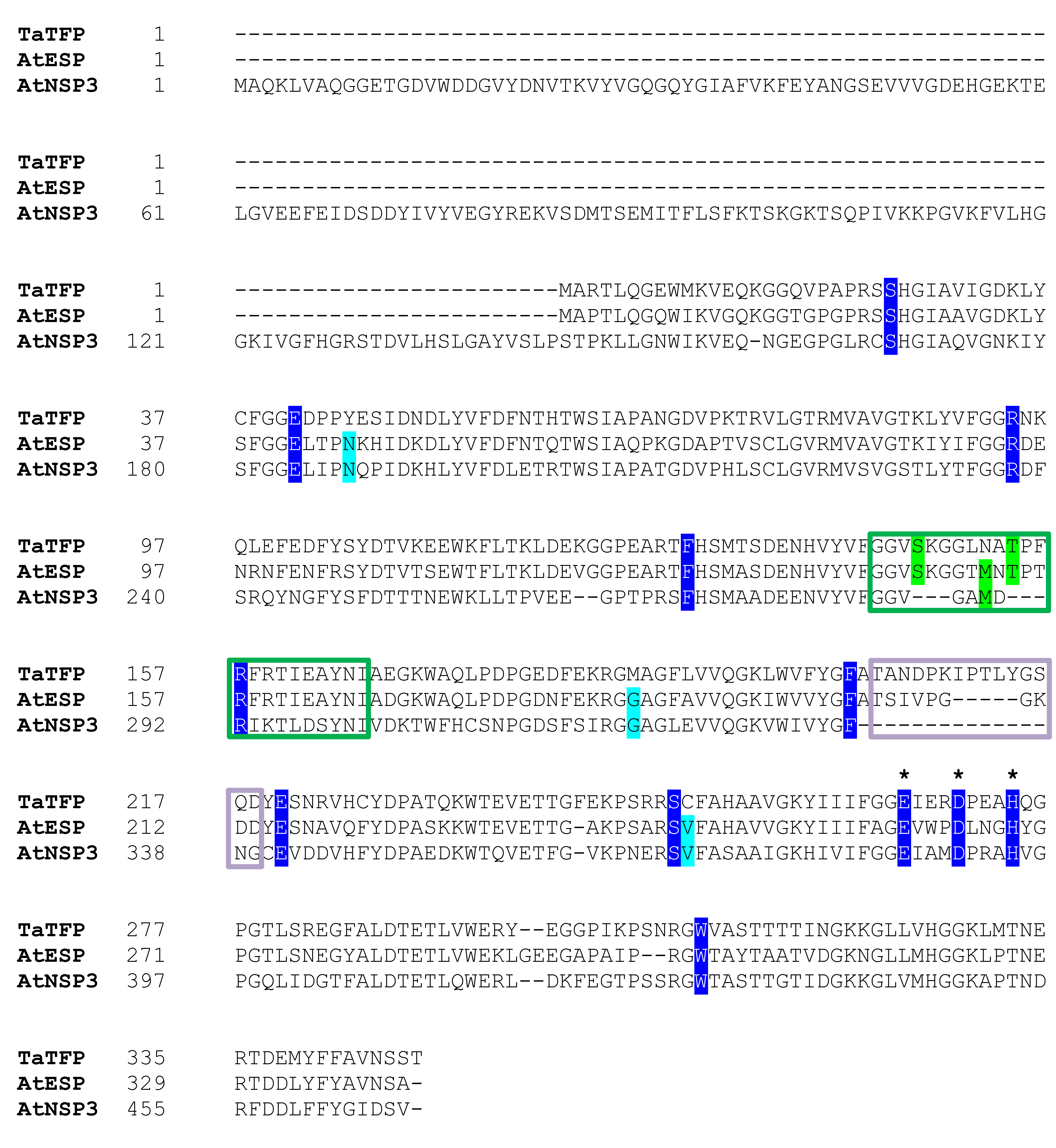

Supplement: Supplementary file 1 — Figure S1. Multiple sequence alignment of TaTFP, AtESP, and AtNSP3. [file TPJ-99-329-s001.tif]

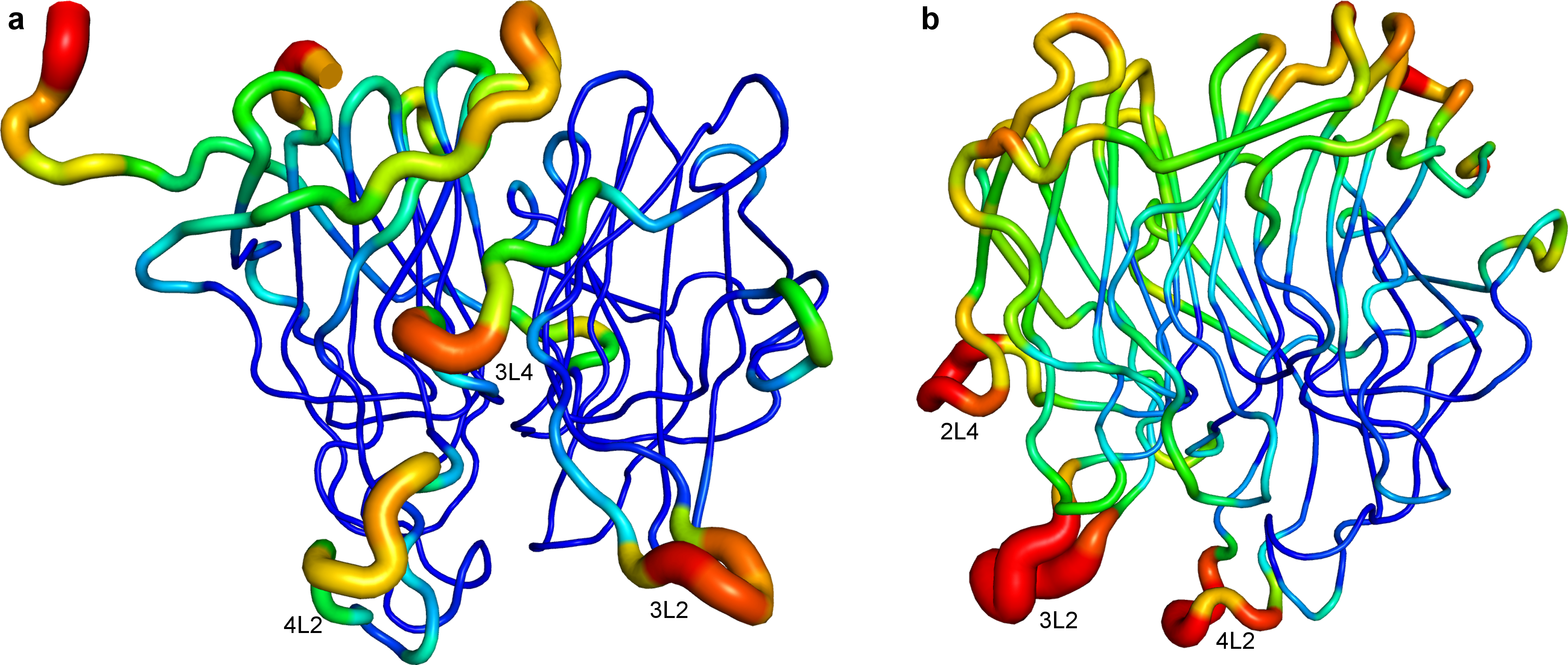

Supplement: Supplementary file 2 — Figure S2. B‐factors of TaTFP and AtESP X‐ray structures. [file TPJ-99-329-s002.tif]

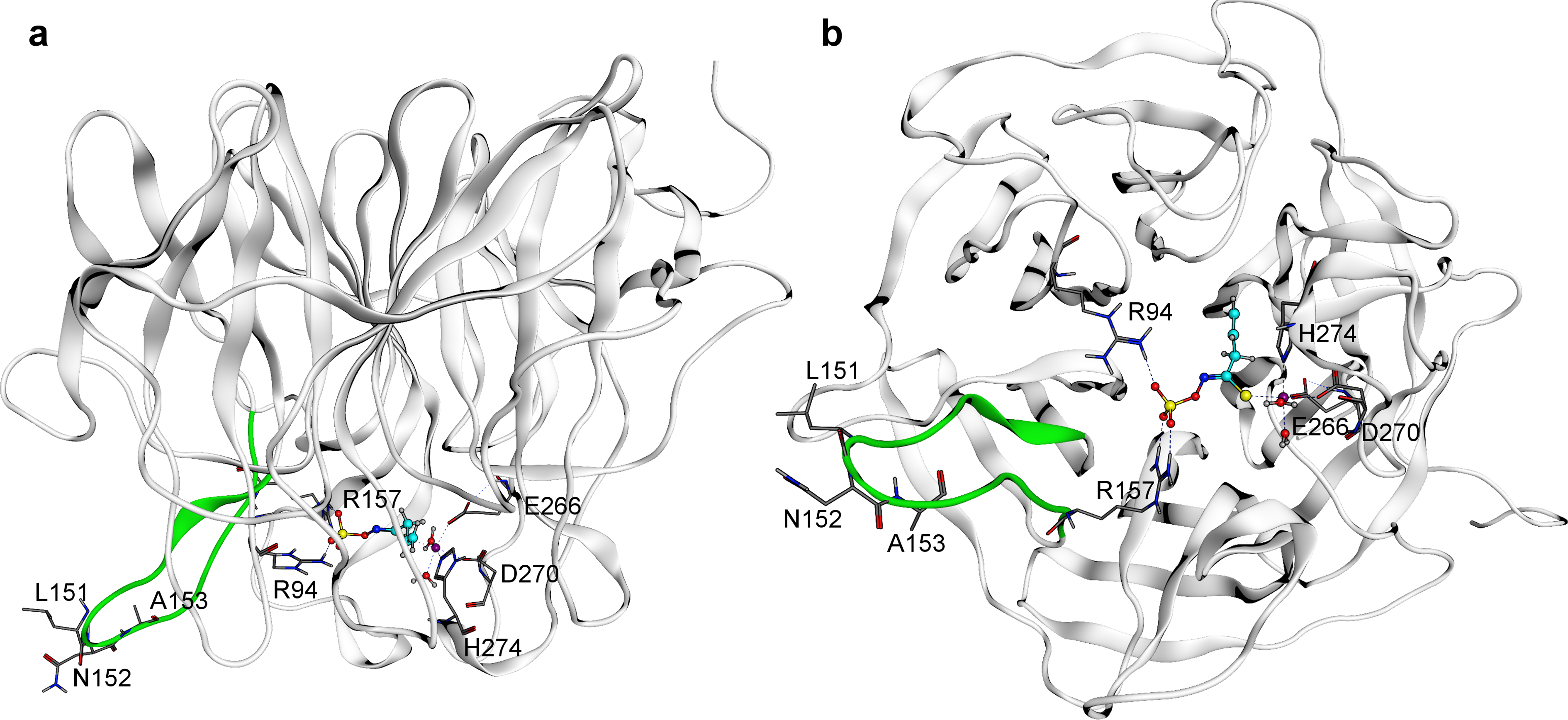

Supplement: Supplementary file 4 — Figure S4. 3L2 conformation of TaTFP X‐ray structure (PDB 5A10). [file TPJ-99-329-s004.tif]
