# Supplementary material for: Structural diversification during glucosinolate breakdown: mechanisms of thiocyanate, epithionitrile and simple nitrile formation
Source: Plant J. 2019 Apr 29;99(2):329–43. doi: 10.1111/tpj.14327 (PMC6850609; doi:10.1111/tpj.14327)
Supplement: Supplementary file 8 — Table S1. Known specifier proteins. [file TPJ-99-329-s008.docx]

**Table S1.** Known specifier proteins. Characterized plant specifier proteins are listed with their UniProt ID and the abbreviation used in the present study.

| **UniProt ID** | **Abbreviation** | **Species** |
| --- | --- | --- |
| J7FPI6 | ApTFP | *Alliaria petiolata* |
| A1XLE2 | LsTFP | *Lepidium sativum* |
| G1FNI6 | TaTFP | *Thlaspi arvense* |
| Q8Ry71 | AtESP | *Arabidopsis thaliana* |
| Q4TU02 | BoESP | *Brassica oleracea var. italica* |
| J7FLJ0 | ChESP | *Cardamine hirsuta* |
| J7FRY5 | CiESP | *Cardamine impatiens* |
| J7FMU8 | DaESP | *Draba aurea* |
| J7FU88 | DlESP | *Draba lanceolata* |
| J7FPI9 | IsESP | *Isatis tinctoria* |
| J7FLJ4 | SpESP | *Schouwia purpurea* |
| Q9SDM9 | AtNSP1 | *Arabidopsis thaliana* |
| O49326 | AtNSP2 | *Arabidopsis thaliana* |
| O04318 | AtNSP3 | *Arabidopsis thaliana* |
| O04316 | AtNSP4 | *Arabidopsis thaliana* |
| Q93XW5 | AtNSP5 | *Arabidopsis thaliana* |
| J7FR70 | ChNSP | *Cardamine hirsuta* |
| J7FMV0 | ItNSP | *Isatis tinctoria* |
| J7FU93 | SpNSP | *Schouwia purpurea* |
