# Supplementary material for: Structural diversification during glucosinolate breakdown: mechanisms of thiocyanate, epithionitrile and simple nitrile formation
Source: Plant J. 2019 Apr 29;99(2):329–43. doi: 10.1111/tpj.14327 (PMC6850609; doi:10.1111/tpj.14327)
Supplement: Supplementary file 9 — Table S2. Primers used for mutagenesis. [file TPJ-99-329-s009.docx]

**Table S2.** Primers used for mutagenesis. Deletion constructs were generated with primers P1-P8. Site-directed mutagenesis was conducted with primers P9-P24 and the corresponding reverse primers (reverse complement sequence). Primers were purchased from Invitrogen/Thermo Fisher Scientific.

| **Nr.** | **Primer** | **Sequence (5'-3')** |
| --- | --- | --- |
| P1 | Thlaspi USER 2 fw. | TAG GCT TAA UAT GGC TCG GAC TTT GC |
| P2 | Thlaspi_USER_rev | TTG GTT TAA UTT ACG TGG AGG AAT TGA CTG C |
| P3 | TaTFP_D204-217.fw | TAC GGA TTC GCG GAC TAC GAG TCT AAT |
| P4 | TaTFP_D204-217.rev | ACG ATT AGA CTC GTA GTC CGC GAA TCC GTA |
| P5 | TaTFP_D210-214.fw | GCG AAT GAT CCT AAG GGG TCC CAG GAC TAC GAG |
| P6 | TaTFP_D210-214.rev | CTC GTA GTC CTG GGA CCC CTT AGG ATC AT |
| P7 | TaTFP_ESP205-217.fw | TCT ATT GTG CCC GGA GGC AAA GAT GAC TAC GAG TCT AAT CGT GTG CA |
| P8 | TaTFP_ESP205-217.rev | ATC TTT GCC TCC GGG CAC AAT AGA AGT CGC GAA TCC GTA AAA CAC CCA |
| P9 | TaTFP_Y45F.fw | GCG AGG ATC CGC CAT TTG AGT CCA TCG AC |
| P10 | TaTFP_Y45N_fw | GAGGATCCGCCAAACGAGTCCATCGAC |
| P11 | TaTFP_L151A_fw | GTG AGC AAA GGA GGG GCG AAT GCA ACC CCC TTT C |
| P12 | TaTFP_L151T_fw | GTG AGC AAA GGA GGG ACG AAT GCA ACC CCC TTT C |
| P13 | TaTFP_N152A_fw | GCA AAG GAG GGC TGG CGG CAA CCC CCT TTC GG |
| P14 | TaTFP_N152M_fw | GAG CAA AGG AGG GCT GAT GGC AAC CCC CTT TCG G |
| P15 | TaTFP_A153N_fw | GCA AAG GAG GGC TGA ATA ATA CCC CCT TTC GGT TCA G |
| P16 | TaTFP_T154A_fw | GGA GGG CTG AAT GCA GCC CCC TTT CGG TTC |
| P17 | TaTFP_LNA151-153TMN | GTG AGC AAA GGA GGC ACG ATG AAT ACC CCC TTT CGG TTC AGG AC |
| P18 | TaTFP_P208A_fw | CGC GAC TGC GAA TGA TGC TAA GAT ACC CAC ACT C |
| P19 | TaTFP_K209A_fw | CGA CTG CGA ATG ATC CTG CGA TAC CCA CAC TC |
| P20 | TaTFP_K209E_fw | CGA CTG CGA ATG ATC CTG AGA TAC CCA CAC TC |
| P21 | TaTFP_T212A_fw | GAT CCT AAG ATA CCC GCA CTC TAC GGG TCC CAG |
| P22 | TaTFP_S216T_fw | CAC ACT CTA CGG GAC CCA GGA CTA CGA GTC |
| P23 | Ta_E272A_fw | GAG CGG GAC CCA GCA GCA CAT CAA GGT C |
| P24 | Ta_A273L_fw | GCG GGA CCC AGA ATT ACA TCA AGG TCC GG |
