# Supplementary material for: Structural diversification during glucosinolate breakdown: mechanisms of thiocyanate, epithionitrile and simple nitrile formation
Source: Plant J. 2019 Apr 29;99(2):329–43. doi: 10.1111/tpj.14327 (PMC6850609; doi:10.1111/tpj.14327)
Supplement: Supplementary file 10 — Appendix S1. Hypothetical explanation for the formation of a racemic mixture of R/S‐3,4‐epithiobutane nitrile by TaTFP [file TPJ-99-329-s010.docx]

**Supporting Information Appendix**

**Appendix S1.** Hypothetical explanation for the formation of a racemic mixture of *R*/*S*-3,4-epithiobutane nitrile by TaTFP

A prerequisite for the formation of both enantiomers seems to be a different side chain orientation of the bound aglucone which enables a reaction between sulfur and the terminal double bond from both sides (Burow, Markert et al. 2006). To predict if TaTFP is capable to produce both enantiomers, the allyl side chain was manually rotated (~ 180° vs. pose I) around the C-C1 bond. After geometry optimization with PM7, the defined active site exhibited marginal conformational changes within the amino acid side chains (pose III, Figure S6a).

The interaction pattern between TaTFP and the aglucone core structure was similar to that of pose I. We hypothesized that pose III might result in formation of *S*-epithionitrile. The heat of formation of pose I is -6.32 kJ mol^‑1^ lower than that of pose III (pose I: ΔH_f_ = ‑5214.10 kJ mol^‑1^; pose III: ΔH_f_ = ‑5207.78 kJ mol^‑1^) indicating a slight preference for the formation of *R*-epithionitrile. To simulate the formation of *S*-epithionitrile, the C-S bond elongation and the distance reduction between S and C3 were defined as reaction coordinates for a semiempirical grid calculation starting with pose III. The reaction process was identical to the formation of *R*‑epithionitrile (Figure 10, Figure S6b). The dissociation of hydrogen sulfate led to an energy release of Δ_r_H_f_ = ‑25.98 kJ mol^‑1^ and resulted in a Fe^2+^/Fe^3+^ oxidation and N radical formation (Figure S6b I to II). Furthermore, the intermediate structure and F202 passed a significant conformational change and enabled a reaction between sulfur and terminal double bond from the other side. The activation barrier to open this bond and to form an S-C2 bond was marginal (E_a_ = 3.35 kJ mol^‑1^, Figure S6b III). The thiirane ring was formed after exceeding the second transition state with an activation energy of E_a_ = 30.54 kJ mol^‑1^ (Figure S6b V) which is 7.11 kJ mol^‑1^ lower than that for the formation of *R*-epithionitrile. Finally, *S*‑epithionitrile was formed. The final heat of formation of the TaTFP active site with bound *S*‑epithionitrile was higher (ΔH_f_ = ‑5422.59 kJ mol^‑1^, Figure S6b VI) than that with bound *R*‑epithionitrile (ΔH_f_ = ‑5465.56 kJ mol^‑1^, Figure 10). Thus, the active site with bound *S*‑epithionitrile is less stable than that with bound *R*-epithionitrile, and *S*-epithionitrile will be released from the active site faster than the *R*-enantiomer.

The entire energy cycles for the formation of *R*‑ and *S*‑epithionitrile were compared in Figure S7. For recovery of TaTFP with protonated R157, which is thermodynamically highly favored (Δ_r_H_f_ = ‑489.74 kJ mol^‑1^), and completion of each energy cycle (Figure S7 (1)) a hydronium ion from the water solution was added.

Enantiomers are generally identical in their heat of formations (ΔH_f_ = 208.74 kJ mol^‑1^ in case of *R*- and *S*-epithionitrile). Moreover, the heat of formation of allylglucosinolate aglucone (ΔH_f_ = ‑549.19 kJ mol^‑1^) and the heat of formation of the free enzyme (TaTFP R157^0^ (ΔH_f_ = ‑4419.28 kJ mol^‑1^) and TaTFP R157^+^ (ΔH_f_ = ‑4056.17 kJ mol^‑1^)) do not depend on either *R*- or *S*-enantiomer formation. Therefore, there is no difference between the resulting complete reaction enthalpies of *R*- vs. *S*-epithionitrile formation. However, next to thermodynamics, kinetics (different activation barriers) could lead to a preference for the formation of one enantiomer.

We have predicted above that the binding of the aglucone in pose I is favored over pose III by  ‑6.32 kJ mol^‑1^ (Δ_r_H_f_ = ‑608.74 kJ mol^‑1^ vs. Δ_r_H_f_ = ‑602.42 kJ mol^‑1^; Figure S7) resulting in a higher concentration of the enzyme-substrate complex of pose I. However, this will be compensated by the lower activation barrier (by 7.11 kJ mol^‑1^) for *S*-epithionitrile formation (30.54 kJ mol^-1^ compared to *R*-epithionitrile 37.65 kJ mol^-1^) and faster release of *S*-epithionitrile from the enzyme (*S*-epithionitrile = 278.54 kJ mol^-1^, *R*-epithionitrile 321.51 kJ mol^‑1^). Thus, the interplay between affinity (thermodynamics) and activation energies (kinetics) may explain the experimentally observed ratio of approximately 1:1.
