# Supplementary material for: Structural diversification during glucosinolate breakdown: mechanisms of thiocyanate, epithionitrile and simple nitrile formation
Source: Plant J. 2019 Apr 29;99(2):329–43. doi: 10.1111/tpj.14327 (PMC6850609; doi:10.1111/tpj.14327)
Supplement: Supplementary file 14 [file TPJ-99-329-s014.docx]

**Full Supporting Information Legends**

**Figure S1.** Multiple sequence alignment of TaTFP, AtESP, and AtNSP3. Fe^2+^ binding triad is marked with asterisks. Conserved active site amino acids are highlighted in dark blue (conservation between the three sequences) and cyan (conservation between two of the three sequences). Amino acids of loops 3L2 and 4L2 of TaTFP are surrounded by a green and lilac box, respectively. The sequence alignment was created with ClustalW (Blosum, gap opening: 10, extending: 0.05, end: 10, separation: 0.05, [www.embnet.vital-it.ch/software/ClustalW.html](http://www.embnet.vital-it.ch/software/ClustalW.html)) and coloring was done manually.

**Figure S2.** B-factors of TaTFP and AtESP X-ray structures. Darkblue indicates small B-factors. Increasing flexibility and B-factor is indicated by warmer colors and increasing thickness of the illustrated structure. **(a)** Most flexible protein structures of TaTFP are the 3L2 and 4L2 loops, and the blade-connecting 3L4 loop. **(b)** In AtESP the 3L2 and 4L2 loop, and the blade-connecting 2L4 loops are the most flexible protein structures. Blade and loop labeling is identical to that in (Gumz, Krausze et al. 2015).

**Figure S3.** Effects of 4L2 loop deletions on TaTFP activity. Mutant proteins and corresponding controls (buffer only (no TFP), wildtype (WT)) were incubated with allylglucosinolate and myrosinase in 50 mM MES buffer, pH 6.0, supplemented with 0.01 mM Fe^2+^ for 40 min. Breakdown products were quantified by GC-FID after dichloromethane extraction. Activity is expressed as the percentage of each product relative to the total amount (nmol) of detected breakdown products. Shown are means ± SD of N=4 independent expression experiments. del, deletion; x, replaced by

**Figure S4.** 3L2 conformation of TaTFP X-ray structure (PDB 5A10). Side view **(a)** and top view **(b)** of the TaTFP with substrate binding site (Gumz, Krausze et al. 2015). Loop 3L2 (green) with the identified L151 N152 A153 triplet. Docked allylglucosinolate aglucone with cyan C-skeleton and Fe^2+^ as purple sphere.

**Figure S5.** *R*/*S*-3,4-epithiobutane nitrile formation in TaTFP-containing reaction mixtures. TaTFP (50 µg) was incubated with allylglucosinolate and myrosinase in 50 mM MES buffer, pH 6.0, supplemented with 0.01 mM Fe^2+^ at 22°C for 40 min. Reaction mixtures were extracted with dichloromethane and analyzed by GC-MS on an FS-Hydrodex ß-6TBDM column (50 m x 0.25 mm x 0.25 µm; Macherey-Nagel, Düren, Germany) using split (1:5) injection and the following temperature program: 35°C for 3 min, 12°C min^-1^ to 85°C hold for 35 min, 10°C min^-1^ to 210°C hold for 10 min. The gas chromatograph was coupled to a mass spectrometer (5975B inert XL EI/CI MSD, Agilent) operated as described previously (Kuchernig, Backenköhler et al. 2011). Products were identified based on their mass spectra (allylisothiocyanate 19.32 min, allylthiocyanate 26.24 min, phenylcyanide 42.37 min, *R*/*S*-3,4-epithiobutane nitrile 49.70 min and 49.76 min).

**Figure S6.** TaTFP-catalyzed formation of *S*-epithionitrile. **(a)** Superposition of pose I and pose III (see Appendix 1). **(b)** Energy profile of *S*‑epithionitrile formation. Numbers on x‑axis are the C‑S and C3‑S distances in Å. Each reaction step is described by corresponding heat of formations. Total reaction enthalpy is Δ_r_H_f_ = ‑214.81 kJ mol^‑1^. **(c)** Optimized geometry of the active site at the final reaction step of *S*-epithionitrile formation. Fe^2+^ purple, oxygen red, sulfur yellow, nitrogen blue, aglucone C-skeleton cyan for *R*‑epithionitrile formation and orange for *S*‑epithionitrile formation, active site amino acid C-skeletons in dark grey for *R*‑epithionitrile formation and in light grey for *S*‑epithionitrile formation.

**Figure S7.** Energy cycles for the formation of *R*- and *S*-epithionitrile. **(1)** Recovery of TaTFP by proton transfer from a hydronium ion to Arg157. **(2)** Formation of TaTFP-aglucone (aglc) complex. **(3)** Predicted catalytic mechanism for *S*‑ (left) and *R*‑epithionitrile (right) formation (details in Figures 10 and S6). **(4)** Product release. Heat of formations of all complexes and compounds (black values), important activation barriers (E_a_) and resulting reaction enthalpies (red values) in kJ mol^‑1^.

**Table S1.** Known specifier proteins. Characterized plant specifier proteins are listed with their UniProt ID and the abbreviation used in the present study.

**Table S2.** Primers used for mutagenesis. Deletion constructs were generated with primers P1-P8. Site-directed mutagenesis was conducted with primers P9-P24 and the corresponding reverse primers (reverse complement sequence). Primers were purchased from Invitrogen/Thermo Fisher Scientific.

**Appendix S1.** Hypothetical explanation for the formation of a racemic mixture of *R*/*S*-3,4-epithiobutane nitrile by TaTFP

**Movie S1.** TaTFP-catalyzed thiocyanate formation.

**Movie S2.** TaTFP-catalyzed epithionitrile formation.

**Movie S3.** AtNSP3-catalyzed allylcyanide formation.
